# Power of data in quantum machine learning

**DOI:** 10.1038/s41467-021-22539-9

**Published:** 2021-05-11

**Authors:** Hsin-Yuan Huang, Michael Broughton, Masoud Mohseni, Ryan Babbush, Sergio Boixo, Hartmut Neven, Jarrod R. McClean

**Affiliations:** 1 Google Quantum AI, Venice, CA USA; 2 Institute for Quantum Information and Matter, Caltech, Pasadena, CA USA; 3 Department of Computing and Mathematical Sciences, Caltech, Pasadena, CA USA

**Keywords:** Computer science, Quantum information

## Abstract

The use of quantum computing for machine learning is among the most exciting prospective applications of quantum technologies. However, machine learning tasks where data is provided can be considerably different than commonly studied computational tasks. In this work, we show that some problems that are classically hard to compute can be easily predicted by classical machines learning from data. Using rigorous prediction error bounds as a foundation, we develop a methodology for assessing potential quantum advantage in learning tasks. The bounds are tight asymptotically and empirically predictive for a wide range of learning models. These constructions explain numerical results showing that with the help of data, classical machine learning models can be competitive with quantum models even if they are tailored to quantum problems. We then propose a projected quantum model that provides a simple and rigorous quantum speed-up for a learning problem in the fault-tolerant regime. For near-term implementations, we demonstrate a significant prediction advantage over some classical models on engineered data sets designed to demonstrate a maximal quantum advantage in one of the largest numerical tests for gate-based quantum machine learning to date, up to 30 qubits.

## Abstract (web summary)

Expectations for quantum machine learning are high, but there is currently a lack of rigorous results on which scenarios would actually exhibit a quantum advantage. Here, the authors show how to tell, for a given dataset, whether a quantum model would give any prediction advantage over a classical one.

## Introduction

As quantum technologies continue to rapidly advance, it becomes increasingly important to understand which applications can benefit from the power of these devices. At the same time, machine learning on classical computers has made great strides, revolutionizing applications in image recognition, text translation, and even physics applications, with more computational power leading to ever increasing performance^1^. As such, if quantum computers could accelerate machine learning, the potential for impact is enormous.

At least two paths towards quantum enhancement of machine learning have been considered. First, motivated by quantum applications in optimization^2–4^, the power of quantum computing could, in principle, be used to help improve the training process of existing classical models^5,6^, or enhance inference in graphical models^7^. This could include finding better optima in a training landscape or finding optima with fewer queries. However, without more structure known in the problem, the advantage along these lines may be limited to quadratic or small polynomial speedups^8,9^.

The second vein of interest is the possibility of using quantum models to generate correlations between variables that are inefficient to represent through classical computation. The recent success both theoretically and experimentally for demonstrating quantum computations beyond classical tractability can be taken as evidence that quantum computers can sample from probability distributions that are exponentially difficult to sample from classically^10,11^. If these distributions were to coincide with real-world distributions, this would suggest the potential for significant advantage. This is typically the type of advantage that has been sought in recent work on both quantum neural networks^12–14^, which seek to parameterize a distribution through some set of adjustable parameters, and quantum kernel methods^15^ that use quantum computers to define a feature map that maps classical data into the quantum Hilbert space. The justification for the capability of these methods to exceed classical models often follows similar lines as refs. ^10,11^ or quantum simulation results. That is, if the model leverages a quantum circuit that is hard to sample results from classically, then there is potential for a quantum advantage.

In this work, we show quantitatively how this picture is incomplete in machine learning (ML) problems where some training data are provided. The provided data can elevate classical models to rival quantum models, even when the quantum circuits generating the data are hard to compute classically. We begin with a motivating example and complexity-theoretic argument showing how classical algorithms with data can match quantum output. Following this, we provide rigorous prediction error bounds for training classical and quantum ML methods based on kernel functions^15–24^ to learn quantum mechanical models. We focus on kernel methods, as they not only provide provable guarantees, but are also very flexible in the functions they can learn. For example, recent advancements in theoretical machine learning show that training neural networks with large hidden layers is equivalent to training an ML model with a particular kernel, known as the neural tangent kernel^19–21^. Throughout, when we refer to classical ML models related to our theoretical developments, we will be referring to ML models that can be easily associated with a kernel, either explicitly as in kernel methods, or implicitly as in the neural tangent kernels. However, in the numerical section, we will also include performance comparisons to methods where direct association of a kernel is challenging, such as random forest methods. In the quantum case, we will also show how quantum ML based on kernels can be made equivalent to training an infinite depth quantum neural network.

We use our prediction error bounds to devise a flowchart for testing potential quantum prediction advantage, the separation between prediction errors of quantum and classical ML models for a fixed amount of training data. The most important test is a geometric difference between kernel functions defined by classical and quantum ML. Formally, the geometric difference is defined by the closest efficient classical ML model. In practice, one should consider the geometric difference with respect to a suite of optimized classical ML models. If the geometric difference is small, then a classical ML method is guaranteed to provide similar or better performance in prediction on the dataset, independent of the function values or labels. Hence this represents a powerful, function independent prescreening that allows one to evaluate if there is any possibility of better performance. On the other hand, if the geometry differs greatly, we show both the existence of a dataset that exhibits large prediction advantage using the quantum ML model and how one can construct it efficiently. While the tools we develop could be used to compare and construct hard classical models like hash functions, we enforce restrictions that allow us to say something about a quantum separation. In particular, the feature map will be white box, in that a quantum circuit specification is available for the ideal feature map, and that feature map can be made computationally hard to evaluate classically. A constructive example of this is a discrete log feature map, where a provable separation for our kernel is given in Supplementary Section 11. Additionally, the minimum over classical models means that classical hash functions are reproduced formally by definition.

Moreover, application of these tools to existing models in the literature rules many of them out immediately, providing a powerful sieve for focusing development of new data encodings. Following these constructions, in numerical experiments, we find that a variety of common quantum models in the literature perform similarly or worse than classical ML on both classical and quantum datasets due to a small geometric difference. The small geometric difference is a consequence of the exponentially large Hilbert space employed by existing quantum models, where all inputs are too far apart. To circumvent the setback, we propose an improvement, which enlarges the geometric difference by projecting quantum states embedded from classical data back to approximate classical representation^25–27^. With the large geometric difference endowed by the projected quantum model, we are able to construct engineered datasets to demonstrate large prediction advantage over common classical ML models in numerical experiments up to 30 qubits. Despite our constructions being based on methods with associated kernels, we find empirically that the prediction advantage remains robust across tested classical methods, including those without an easily determined kernel. This opens the possibility to use a small quantum computer to generate efficiently verifiable machine learning problems that could be challenging for classical ML models.

## Results

### Setup and motivating example

We begin by setting up the problems and methods of interest for classical and quantum models, and then provide a simple motivating example for studying how data can increase the power of classical models on quantum data. The focus will be a supervised learning task with a collection of *N* training examples {(*x*_*i*_, *y*_*i*_)}, where *x*_*i*_ is the input data and *y*_*i*_ is an associated label or value. We assume that *x*_*i*_ are sampled independently from a data distribution ${\mathcal{D}}$.

In our theoretical analysis, we will consider ${y}_{i}\in {\mathbb{R}}$ to be generated by some quantum model. In particular, we consider a continuous encoding unitary that maps a classical input data *x*_*i*_ into quantum state $\left|{x}_{i}\right\rangle ={U}_{\text{enc}}({x}_{i}){\left|0\right\rangle }^{\otimes n}$ and refer to the corresponding density matrix as *ρ*(*x*_*i*_). The expressive power of these embeddings have been investigated from a functional analysis point of view^28,29^; however, the setting where data are provided requires special attention. The encoding unitary is followed by a unitary *U*_QNN_(*θ*). We then measure an observable *O* after the quantum neural network. This produces the label/value for input *x*_*i*_ given as ${y}_{i}=f({x}_{i})=\left\langle {x}_{i}\right|{U}_{\,\text{QNN}}^{\dagger }O{U}_{\text{QNN}}\left|{x}_{i}\right\rangle$. The quantum model considered here is also referred to as a quantum neural network (QNN) in the literature^14,30^. The goal is to understand when it is easy to predict the function *f*(*x*) by training classical/quantum machine learning models.

With notation in place, we turn to a simple motivating example to understand how the availability of data in machine learning tasks can change computational hardness. Consider data points ${\{{{\bf{x}}}_{i}\}}_{i = 1}^{N}$ that are *p*-dimensional classical vectors with ∣∣**x**_*i*_∣∣_2_ = 1, and use amplitude encoding^31–33^ to encode the data into an *n*-qubit state $\left|{{\bf{x}}}_{i}\right\rangle =\mathop{\sum }\nolimits_{k = 1}^{p}{x}_{i}^{k}\left|k\right\rangle$, where ${x}_{i}^{k}$ is the individual coordinate of the vector **x**_*i*_. If *U*_QNN_ is a time-evolution under a many-body Hamiltonian, then the function $f({\bf{x}})=\left\langle {\bf{x}}\right|{U}_{\,\text{QNN}}^{\dagger }O{U}_{\text{QNN}}\left|{\bf{x}}\right\rangle$ is in general hard to compute classically^34^, even for a single input state. In particular, we have the following proposition showing that if a classical algorithm can compute *f*(**x**) efficiently, then quantum computers will be no more powerful than classical computers; see Supplementary Section 1 for a proof.

### Proposition 1

If a classical algorithm without training data can compute *f*(**x**) efficiently for any *U*_QNN_ and *O*, then BPP = BQP.

Nevertheless, it is incorrect to conclude that training a classical model from data to learn this evolution is hard. To see this, we write out the expectation value as

1 $$f({x}_{i})\,=\,	\left(\mathop{\sum }\limits_{k=1}^{p}{x}_{i}^{k* }\left\langle k\right|\right){U}_{\,\text{QNN}}^{\dagger }O{U}_{\text{QNN}}\left(\mathop{\sum }\limits_{l=1}^{p}{x}_{i}^{l}\left|l\right\rangle \right)\\ =\,	\mathop{\sum }\limits_{k=1}^{p}\mathop{\sum }\limits_{l=1}^{p}{B}_{kl}{x}_{i}^{k* }{x}_{i}^{l},$$

which is a quadratic function with *p*^2^ coefficients ${B}_{kl}=\left\langle k\right|{U}_{\,\text{QNN}}^{\dagger }O{U}_{\text{QNN}}\left|l\right\rangle$. Using the theory developed later in this work, we can show that, for any *U*_QNN_ and *O*, training a specific classical ML model on a collection of *N* training examples {(**x**_*i*_, *y*_*i*_ = *f*(**x**_*i*_))} would give rise to a prediction model *h*(**x**_*i*_) with

2 $${{\mathbb{E}}}_{{\bf{x}} \sim {\mathcal{D}}}| h({\bf{x}})-f({\bf{x}})| \le c\sqrt{\frac{{p}^{2}}{N}},$$

for a constant *c* > 0. We refer to Supplementary Section 1 for the proof of this result. Hence, with *N* ∝ *p*^2^/*ϵ*^2^ training data, one can train a classical ML model to predict the function *f*(**x**) up to an additive prediction error *ϵ*. This elevation of classical models through some training samples is illustrative of the power of data. In Supplementary Section 2, we give a rigorous complexity-theoretic argument on the computational power provided by data. A cartoon depiction of the complexity separation induced by data is provided in Fig. 1(a).

**Fig. 1:**
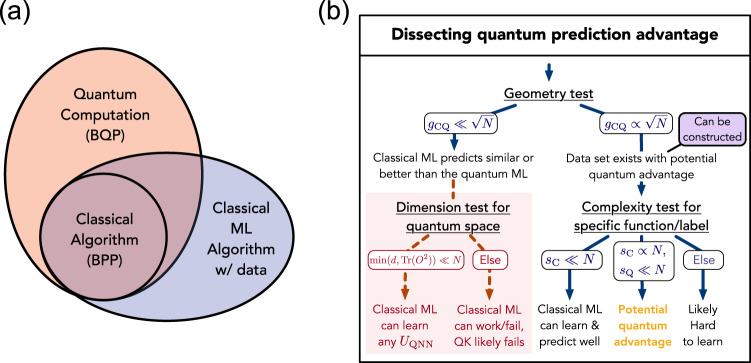
Illustration of the relation between complexity classes and a flowchart for understanding and prescreening potential quantum advantage. **a** We cartoon the separation between problem complexities that are created by the addition of data to a problem. Classical algorithms that can learn from data define a complexity class that can solve problems beyond classical computation (BPP), but it is still expected that quantum computation can efficiently solve problems that classical ML algorithms with data cannot. A rigorous definition and proof for the separation between classical algorithms that can learn from data and BPP/BQP is given in Supplementary Section 2. **b** The flowchart we develop for understanding the potential for quantum prediction advantage. *N* samples of data from a potentially infinite depth QNN made with encoding and function circuits *U*_enc_ and *U*_QNN_ are provided as input along with quantum and classical methods with associated kernels. Tests are given as functions of *N* to emphasize the role of data in the possibility of a prediction advantage. One can first evaluate a geometric quantity *g*_CQ_ that measures the possibility of an advantageous quantum/classical prediction separation without yet considering the actual function to learn. We show how one can efficiently construct an adversarial function that saturates this limit if the test is passed, otherwise the classical approach is guaranteed to match performance for any function of the data. To subsequently consider the actual function provided, a label/function-specific test may be run using the model complexities *s*_*C*_ and *s*_*Q*_. If one specifically uses the quantum kernel (QK) method, the red dashed arrows can evaluate if all possible choices of *U*_QNN_ lead to an easy classical function for the chosen encoding of the data.

While this simple example makes the basic point that sufficient data can change complexity considerations, it perhaps opens more questions than it answers. For example, it uses a rather weak encoding into amplitudes and assumes one has access to an amount of data that is on par with the dimension of the model. The more interesting cases occur if we strengthen the data encoding, include modern classical ML models, and consider the number of data *N* much less than the dimension of the model. These more interesting cases are the ones we quantitatively answer.

Our primary interest will be ML algorithms that are much stronger than fitting a quadratic function and the input data are provided in more interesting ways than an amplitude encoding. In this work, we focus on both classical and quantum ML models based on kernel functions *k*(*x*_*i*_, *x*_*j*_). At a high level, a kernel function can be seen as a measure of similarity, if *k*(*x*_*i*_, *x*_*j*_) is large when *x*_*i*_ and *x*_*j*_ are close. When considered for finite input data, a kernel function may be represented as a matrix *K*_*ij*_ = *k*(*x*_*i*_, *x*_*j*_) and the conditions required for kernel methods are satisfied when the matrix representation is Hermitian and positive semi-definite.

A given kernel function corresponds to a nonlinear feature mapping *ϕ*(*x*) that maps *x* to a possibly infinite-dimensional feature space, such that $k({x}_{i},{x}_{j})=\phi {({x}_{i})}^{\dagger }\phi ({x}_{j})$. This is the basis of the so-called “kernel trick” where intricate and powerful maps *ϕ*(*x*_*i*_) can be implemented through the evaluation of relatively simple kernel functions *k*. As a simple case, in the example above, using a kernel of *k*(*x*_*i*_, *x*_*j*_) = ∣〈*x*_*i*_∣*x*_*j*_〉∣^2^ corresponds to a feature map $\phi ({x}_{i})={\sum }_{kl}{x}_{i}^{k* }{x}_{i}^{l}\left|k\right\rangle \otimes \left|l\right\rangle$ which is capable of learning quadratic functions in the amplitudes. In kernel based ML algorithms, the trained model can always be written as *h*(*x*) = **w**^†^*ϕ*(*x*) where **w** is a vector in the feature space defined by the kernel. For example, training a convolutional neural network with large hidden layers^19,35^ is equivalent to using a corresponding neural tangent kernel *k*^CNN^. The feature map *ϕ*^CNN^ for the kernel *k*^CNN^ is a nonlinear mapping that extracts all local properties of *x*^35^. In quantum mechanics, similarly a kernel function can be defined using the native geometry of the quantum state space $\left|x\right\rangle$. For example, we can define the kernel function as 〈*x*_*i*_∣*x*_*j*_〉 or ∣〈*x*_*i*_∣*x*_*j*_〉∣^2^. Using the output from this kernel in a method like a classical support vector machine^16^ defines the quantum kernel method.

A wide class of functions can be learned with a sufficiently large amount of data by using the right kernel function *k*. For example, in contrast to the perhaps more natural kernel, 〈*x*_*i*_∣*x*_*j*_〉, the quantum kernel *k*^Q^(*x*_*i*_, *x*_*j*_) = ∣〈*x*_*i*_∣*x*_*j*_〉∣^2^ = Tr(*ρ*(*x*_*i*_)*ρ*(*x*_*j*_)) can learn arbitrarily deep quantum neural network *U*_QNN_ that measures any observable *O* (shown in Supplementary Section 3), and the Gaussian kernel, ${k}^{\gamma }({x}_{i},{x}_{j})=\exp (-\gamma | | {x}_{i}-{x}_{j}| {| }^{2})$ with hyperparameter *γ*, can learn any continuous function in a compact space^36^, which includes learning any QNN. Nevertheless, the required amount of data *N* to achieve a small prediction error could be very large in the worst case. Although we will work with other kernels defined through a quantum space, due both to this expressive property and terminology of past work, we will refer to ${k}^{{\rm{Q}}}({x}_{i},{x}_{j})=\,\text{Tr}\,\big[\rho ({x}_{i})\rho ({x}_{j})\big]$ as the quantum kernel method throughout this work, which is also the definition given in^15^.

### Testing quantum advantage

We now construct our more general framework for assessing the potential for quantum prediction advantage in a machine learning task. Beginning from a general result, we build both intuition and practical tests based on the geometry of the learning spaces. This framework is summarized in Fig. 1.

Our foundation is a general prediction error bound for training classical/quantum ML models to predict some quantum model defined by *f*(*x*) = Tr(*O*^*U*^*ρ*(*x*)) derived from concentration inequalities, where ${O}^{U}={U}_{\,\text{QNN}}^{\dagger }O{U}_{\text{QNN}}$. Suppose we have obtained *N* training examples {(*x*_*i*_, *y*_*i*_ = *f*(*x*_*i*_))}. After training on this data, there exists an ML algorithm that outputs *h*(*x*) = **w**^†^*ϕ*(*x*) using kernel $k({x}_{i},{x}_{j})={K}_{ij}=\phi {({x}_{i})}^{\dagger }\phi ({x}_{j})$, which has a simplified prediction error bounded by

3 $${{\mathbb{E}}}_{x \sim {\mathcal{D}}}| h(x)-f(x)| \le c\sqrt{\frac{{s}_{K}(N)}{N}}$$

for a constant *c* > 0 and *N* independent samples from the data distribution ${\mathcal{D}}$. We note here that this and all subsequent bounds have a key dependence on the quantity of data *N*, reflecting the role of data to improve prediction performance. Due to a scaling freedom between *αϕ*(*x*) and **w**/*α*, we have assumed $\mathop{\sum }\nolimits_{i = 1}^{N}\phi {({x}_{i})}^{\dagger }\phi ({x}_{i})=\,\text{Tr}\,(K)=N$. A derivation of this result is given in Supplementary Section 4.

Given this core prediction error bound, we now seek to understand its implications. The main quantity that determines the prediction error is

4 $${s}_{K}(N)=\mathop{\sum }\limits_{i=1}^{N}\mathop{\sum }\limits_{j=1}^{N}{({K}^{-1})}_{ij}\,\text{Tr}\,({O}^{U}\rho ({x}_{i}))\,\text{Tr}\,({O}^{U}\rho ({x}_{j})).$$

The quantity *s*_*K*_(*N*) is equal to the model complexity of the trained function *h*(*x*) = **w**^†^*ϕ*(*x*), where *s*_*K*_(*N*) = ∣∣**w**∣∣^2^ = **w**^†^**w** after training. A smaller value of *s*_*K*_(*N*) implies better generalization to new data *x* sampled from the distribution ${\mathcal{D}}$. Intuitively, *s*_*K*_(*N*) measures whether the closeness between *x*_*i*_, *x*_*j*_ defined by the kernel function *k*(*x*_*i*_, *x*_*j*_) matches well with the closeness of the observable expectation for the quantum states *ρ*(*x*_*i*_), *ρ*(*x*_*j*_), recalling that a larger kernel value indicates two points are closer. The computation of *s*_*K*_(*N*) can be performed efficiently on a classical computer by inverting an *N* × *N* matrix *K* after obtaining the *N* values Tr(*O*^*U*^*ρ*(*x*_*i*_)) by performing order *N* experiments on a physical quantum device. The time complexity scales at most as order *N*^3^. Due to the connection between **w**^†^**w** and the model complexity, a regularization term **w**^†^**w** is often added to the optimization problem during the training of *h*(*x*) = **w**^†^*ϕ*(*x*), see e.g., refs. ^16,37,38^. Regularization prevents *s*_*K*_(*N*) from becoming too large at the expense of not completely fitting the training data. A detailed discussion and proof under regularization is given in Supplementary Section 4 and 6.

The prediction error upper bound can often be shown to be asymptotically tight by proving a matching lower bound. As an example, when *k*(*x*_*i*_, *x*_*j*_) is the quantum kernel Tr(*ρ*(*x*_*i*_)*ρ*(*x*_*j*_)), we can deduce that *s*_*K*_(*N*) ≤ Tr(*O*^2^) hence one would need a number of data *N* scaling as Tr(*O*^2^). In Supplementary Section 8, we give a matching lower bound showing that a scaling of Tr(*O*^2^) is unavoidable if we assume a large Hilbert space dimension. This lower bound holds for any learning algorithm and not only for quantum kernel methods. The lower bound proof uses mutual information analysis and could easily extend to other kernels. This proof strategy is also employed extensively in a follow-up work^39^ to devise upper and lower bounds for classical and quantum ML in learning quantum models. Furthermore, not only are the bounds asymptotically tight, in numerical experiments given in Supplementary Section 13 we find that the prediction error bound also captures the performance of other classical ML models not based on kernels where the constant factors are observed to be quite modest.

Given some set of data, if *s*_*K*_(*N*) is found to be small relative to *N* after training for a classical ML model, this quantum model *f*(*x*) can be predicted accurately even if *f*(*x*) is hard to compute classically for any given *x*. In order to formally evaluate the potential for quantum prediction advantage generally, one must take *s*_*K*_(*N*) to be the minimal over efficient classical models. However, we will be more focused on minimally attainable values over a reasonable set of classical methods with tuned hyperparameters. This prescribes an effective method for evaluating potential quantum advantage in practice, and already rules out a considerable number of examples from the literature.

**Fig. 2:**
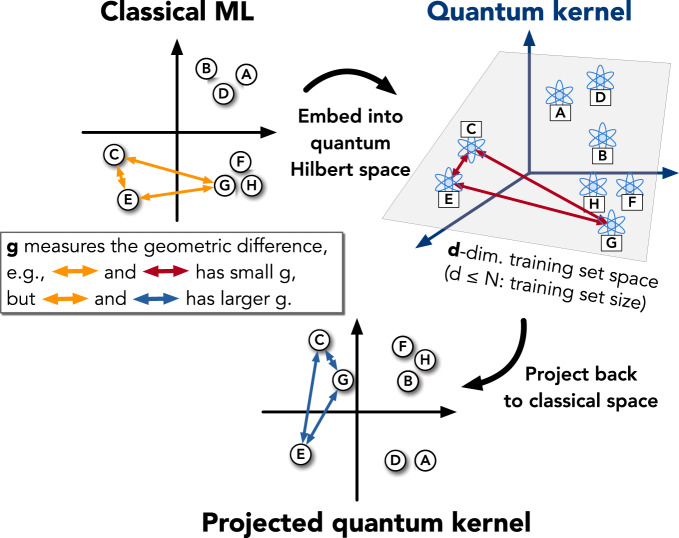
Cartoon of the geometry (kernel function) defined by classical and quantum ML models. The letters A, B, ... represent data points {*x*_*i*_} in different spaces with arrows representing the similarity measure (kernel function) between data. The geometric difference *g* is a difference between similarity measures (arrows) in different ML models and *d* is an effective dimension of the dataset in the quantum Hilbert space.

From the bound, we can see that the potential advantage for one ML algorithm defined by *K*^1^ to predict better than another ML algorithm defined by *K*^2^ depends on the largest possible separation between ${s}_{{K}^{1}}$ and ${s}_{{K}^{2}}$ for a dataset. The separation can be characterized by defining an asymmetric geometric difference that depends on the dataset, but is independent of the function values or labels. Hence evaluating this quantity is a good first step in understanding if there is a potential for quantum advantage, as shown in Fig. 1. This quantity is defined by

5 $${g}_{12}=g({K}^{1}| | {K}^{2})=\sqrt{| | \sqrt{{K}^{2}}{({K}^{1})}^{-1}\sqrt{{K}^{2}}| {| }_{\infty }},$$

where ∣∣. ∣∣_*∞*_ is the spectral norm of the resulting matrix and we assume Tr(*K*^1^) = Tr(*K*^2^) = *N*. One can show that ${s}_{{K}^{1}}\le {g}_{12}^{2}{s}_{{K}^{2}}$, which implies the prediction error bound $c\sqrt{{s}_{{K}^{1}}/N}\le c{g}_{12}\sqrt{{s}_{{K}^{2}}/N}$. A detailed derivation is given in Supplementary Section C and an illustration of *g*_12_ can be found in Fig. 2. The geometric difference *g*(*K*^1^∣∣*K*^2^) can be computed on a classical computer by performing a singular value decomposition of the *N* × *N* matrices *K*^1^ and *K*^2^. Standard numerical analysis packages^40^ provide highly efficient computation of a singular value decomposition in time at most order *N*^3^. Intuitively, if *K*^1^(*x*_*i*_, *x*_*j*_) is small/large when *K*^2^(*x*_*i*_, *x*_*j*_) is small/large, then the geometric difference *g*_12_ is a small value ~1, where *g*_12_ grows as the kernels deviate.

To see more explicitly how the geometric difference allows one to make statements about the possibility for one ML model to make different predictions from another, consider the geometric difference *g*_CQ_ = *g*(*K*^C^∣∣*K*^Q^) between a classical ML model with kernel *k*^C^(*x*_*i*_, *x*_*j*_) and a quantum ML model, e.g., with *k*^Q^(*x*_*i*_, *x*_*j*_) = Tr(*ρ*(*x*_*i*_)*ρ*(*x*_*j*_)). If *g*_CQ_ is small, because

6 $${s}_{{\rm{C}}}\le {g}_{{\rm{CQ}}}^{2}{s}_{{\rm{Q}}},$$

the classical ML model will always have a similar or better model complexity *s*_*K*_(*N*) compared to the quantum ML model. This implies that the prediction performance for the classical ML will likely be competitive or better than the quantum ML model, and one is likely to prefer using the classical model. This is captured in the first step of our flowchart in Fig. 1.

In contrast, if *g*_CQ_ is large we show that there exists a dataset with ${s}_{{\rm{C}}}={g}_{{\rm{CQ}}}^{2}{s}_{{\rm{Q}}}$ with the quantum model exhibiting superior prediction performance. An efficient method to explicitly construct such a maximally divergent dataset is given in Supplementary Section 7 and a numerical demonstration of the stability of this separation is provided in the next section. While a formal statement about classical methods generally requires defining it overall efficient classical methods, in practice, we consider *g*_CQ_ to be the minimum geometric difference among a suite of optimized classical ML models. Our engineered approach minimizes this value as a hyperparameter search to find the best classical adversary, and shows remarkable robustness across classical methods including those without an associated kernel, such as random forests^41^.

In the specific case of the quantum kernel method with ${K}_{ij}^{Q}={k}^{{\rm{Q}}}({x}_{i},{x}_{j})=\,\text{Tr}\,(\rho ({x}_{i})\rho ({x}_{j}))$, we can gain additional insights into the model complexity *s*_*K*_, and sometimes make conclusions about classically learnability for all possible *U*_QNN_ for the given encoding of the data. Let us define vec(*X*) for a Hermitian matrix *X* to be a vector containing the real and imaginary part of each entry in *X*. In this case, we find ${s}_{Q}={\rm{vec}}{({O}^{U})}^{T}{P}_{Q}{\rm{vec}}({O}^{U})$, where *P*_*Q*_ is the projector onto the subspace formed by {vec(*ρ*(*x*_1_)), …, vec(*ρ*(*x*_*N*_))}. We highlight

7 $$d=\,{\text{dim}}\,({P}_{Q})=\,{\text{rank}}\,({K}^{{\rm{Q}}})\le N,$$

which defines the effective dimension of the quantum state space spanned by the training data. An illustration of the dimension *d* can be found in Fig. 1. Because *P*_*Q*_ is a projector and has eigenvalues 0 or 1, ${s}_{{\rm{Q}}}\le \min (d,{\rm{vec}}{({O}^{U})}^{T}{\rm{vec}}({O}^{U}))=\min (d,\,\text{Tr}\,({O}^{2}))$ assuming ∣∣*O*∣∣_*∞*_ ≤ 1. Hence in the case of the quantum kernel method, the prediction error bound may be written as

8 $${{\mathbb{E}}}_{x\in {\mathcal{D}}}| h(x)-f(x)| \le c\sqrt{\frac{\min (d,\,\text{Tr}\,({O}^{2}))}{N}}.$$

A detailed derivation is given in Supplementary Section A. We can also consider the approximate dimension *d*, where small eigenvalues in *K*^Q^ are truncated, by incurring a small training error. After obtaining *K*^Q^ from a quantum device, the dimension *d* can be computed efficiently on a classical machine by performing a singular value decomposition on the *N* × *N* matrix *K*^Q^. Estimation of Tr(*O*^2^) can be performed by sampling random states $\left|\psi \right\rangle$ from a quantum 2-design, measuring *O* on $\left|\psi \right\rangle$, and performing statistical analysis on the measurement data^25^. This prediction error bound shows that a quantum kernel method can learn any *U*_QNN_ when the dimension of the training set space *d* or the squared Frobenius norm of observable Tr(*O*^2^) is much smaller than the amount of data *N*. In Supplementary Section 8, we show that quantum kernel methods are optimal for learning quantum models with bounded Tr(*O*^2^) as they saturate the fundamental lower bound. However, in practice, most observables, such as Pauli operators, will have exponentially large Tr(*O*^2^), so the central quantity is the dimension *d*. Using the prediction error bound for the quantum kernel method, if both *g*_CQ_ and $\min (d,\,\text{Tr}\,({O}^{2}))$ are small, then a classical ML would also be able to learn any *U*_QNN_. In such a case, one must conclude that the given encoding of the data is classically easy, and this cannot be affected by an arbitrarily deep *U*_QNN_. This constitutes the bottom left part of our flowchart in Fig. 1.

Ultimately, to see a prediction advantage in a particular dataset with specific function values/labels, we need a large separation between *s*_C_ and *s*_Q_. This happens when the inputs *x*_*i*_, *x*_*j*_ considered close in a quantum ML model are actually close in the target function *f*(*x*), but are far in classical ML. This is represented as the final test in Fig. 1 and the methodology here outlines how this result can be achieved in terms of its more essential components.

### Projected quantum kernels

In addition to analyzing existing quantum models, the analysis approach introduced also provides suggestions for new quantum models with improved properties, which we now address here. For example, if we start with the original quantum kernel, when the effective dimension *d* is large, kernel Tr(*ρ*(*x*_*i*_)*ρ*(*x*_*j*_)), which is based on a fidelity-type metric, will regard all data to be far from each other and the kernel matrix *K*^Q^ will be close to identity. This results in a small geometric difference *g*_CQ_ leading to classical ML models being competitive or outperforming the quantum kernel method. In Supplementary Section 9, we present a simple quantum model that requires an exponential amount of samples to learn using the quantum kernel Tr(*ρ*(*x*_*i*_)*ρ*(*x*_*j*_)), but only needs a linear number of samples to learn using a classical ML model.

To circumvent this setback, we propose a family of projected quantum kernels as a solution. These kernels work by projecting the quantum states to an approximate classical representation, e.g., using reduced physical observables or classical shadows^25,27,42–44^. Even if the training set space has a large dimension *d* ~ *N*, the projection allows us to reduce to a low-dimensional classical space that can generalize better. Furthermore, by going through the exponentially large quantum Hilbert space, the projected quantum kernel can be challenging to evaluate without a quantum computer. In numerical experiments, we find that the classical projection increases rather than decreases the geometric difference with classical ML models. These constructions will be the foundation of our best performing quantum method later.

One of the simplest forms of projected quantum kernel is to measure the one-particle reduced density matrix (1-RDM) on all qubits for the encoded state, *ρ*_*k*_(*x*_*i*_) = Tr_*j*≠*k*_[*ρ*(*x*_*i*_)], then define the kernel as

9 $${k}^{\text{PQ}}({x}_{i},{x}_{j})=\exp \left(-\gamma \mathop{\sum}\limits_{k}| | {\rho }_{k}({x}_{i})-{\rho }_{k}({x}_{j})| {| }_{F}^{2}\right).$$

This kernel defines a feature map function in the 1-RDM space that is capable of expressing arbitrary functions of powers of the 1-RDMs of the quantum state. From nonintuitive results in density functional theory, we know even one body densities can be sufficient for determining exact ground state^45^ and time-dependent^46^ properties of many-body systems under modest assumptions. In Supplementary Section 10, we provide examples of other projected quantum kernels. This includes an efficient method for computing a kernel function that contains all orders of RDMs using local randomized measurements and the formalism of classical shadows^25^. The classical shadow formalism allows efficient construction of RDMs from very few measurements. In Supplementary Section 11, we show that projected versions of quantum kernels lead to a simple and rigorous quantum speed-up in a recently proposed learning problem based on discrete logarithms^24^.

### Numerical studies

We now provide numerical evidence up to 30 qubits that supports our theory on the relation between the dimension *d*, the geometric difference *g*, and the prediction performance. Using the projected quantum kernel, the geometric difference *g* is much larger and we see the strongest empirical advantage of a scalable quantum model on quantum datasets to date. These are the largest combined simulation and analysis in digital quantum machine learning that we are aware of, and make use of the TensorFlow and TensorFlow-Quantum package^47^, reaching a peak throughput of up to 1.1 quadrillion floating point operations per second (petaflop/s). Trends of ~300 teraflop/s for quantum simulation and 800 teraflop/s for classical analysis were observed up to the maximum experiment size with the overall floating point operations across all experiments totalling ~2 quintillion (exaflop).

In order to mimic a data distribution that pertains to real-world data, we conduct our experiments around the fashion-MNIST dataset^48^, which is an image classification for distinguishing clothing items, and is more challenging than the original digit-based MNIST source^49^. We preprocess the data using principal component analysis^50^ to transform each image into an *n*-dimensional vector. The same data are provided to the quantum and classical models, where in the classical case the data is the *n*-dimensional input vector, and the quantum case uses a given circuit to embed the *n*-dimensional vector into the space of *n* qubits. For quantum embeddings, we explore three options, E1 is a separable rotation circuit^32,51,52^, E2 is an IQP-type embedding circuit^15^, and E3 is a Hamiltonian evolution circuit, with explicit constructions in Supplementary Section 12.

For the classical ML task (C), the goal is to correctly identify the images as shirts or dresses from the original dataset. For the quantum ML tasks, we use the same fashion-MINST source data and embeddings as above, but take as function values the expectation value of a local observable that has been evolved under a quantum neural network resembling the Trotter evolution of 1D-Heisenberg model with random couplings. In these cases, the embedding is taken as part of the ground truth, so the resulting function will be different depending on the quantum embedding. For these ML tasks, we compare against the best performing model from a list of standard classical ML algorithms with properly tuned hyperparameters (see Supplementary Section 12 for details).

**Fig. 3:**
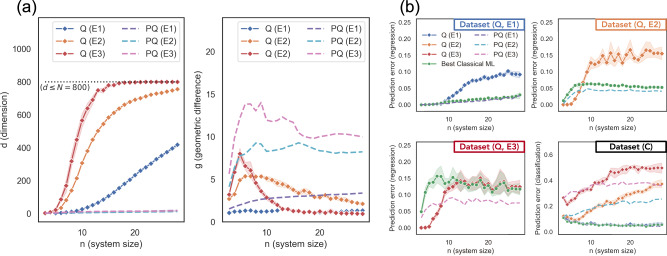
Relation between dimension *d*, geometric difference *g*, and prediction performance. The shaded regions are the standard deviation over 10 independent runs and *n* is the number of qubits in the quantum encoding and dimension of the input for the classical encoding. **a** The approximate dimension *d* and the geometric difference *g* with classical ML models for quantum kernel (Q) and projected quantum kernel (PQ) under different embeddings and system sizes *n*. **b** Prediction error (lower is better) of the quantum kernel method (Q), projected quantum kernel method (PQ), and classical ML models on classical (C) and quantum (Q) datasets with number of data *N* = 600. As *d* grows too large, the geometric difference *g* for quantum kernel becomes small. We see that small geometric difference *g* always results in classical ML being competitive or outperforming the quantum ML model. When *g* is large, there is a potential for improvement over classical ML. For example, projected quantum kernel improves upon the best classical ML in Dataset (Q, E3).

**Fig. 4:**
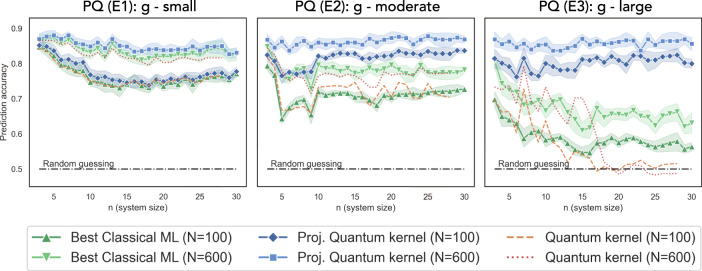
Prediction accuracy (higher the better) on engineered datasets. A label function is engineered to match the geometric difference *g*(C∣∣PQ) between projected quantum kernel and classical approaches, demonstrating a significant gap between quantum and the best classical models up to 30 qubits when *g* is large. We consider the best performing classical ML models among Gaussian SVM, linear SVM, Adaboost, random forest, neural networks, and gradient boosting. We only report the accuracy of the quantum kernel method up to system size *n* = 28 due to the high simulation cost and the inferior performance.

In Fig. 3, we give a comparison between the prediction performance of classical and quantum ML models. One can see that not only do classical ML models perform best on the original classical dataset, the prediction performance for the classical methods on the quantum datasets is also very competitive and can even outperform existing quantum ML models despite the quantum ML models having access to the training embedding while the classical methods do not. The performance of the classical ML model is especially strong on Dataset (Q, E1) and Dataset (Q, E2). This elevation of the classical performance is evidence of the power of data. Moreover, this intriguing behavior and the lack of quantum advantage may be explained by considering the effective dimension *d* and the geometric difference *g* following our theoretical constructions. From Fig. 3a, we can see that the dimension *d* of the original quantum state space grows rather quickly, and the geometric difference *g* becomes small as the dimension becomes too large (*d* ∝ *N*) for the standard quantum kernel. The saturation of the dimension coincides with the decreasing and statistical fluctuations in performance seen in Fig. 4. Moreover, given poor ML performance a natural instinct is to throw more resources at the problem, e.g., more qubits, but as demonstrated here, doing this for naïve quantum kernel methods is likely to lead to tiny inner products and even worse performance. In contrast, the projected quantum space has a low dimension even when *d* grows, and yields a higher geometric difference *g* for all embeddings and system sizes. Our methodology predicts that, when *g* is small, classical ML model will be competitive or outperform the quantum ML model. This is verified in Fig. 3b for both the original and projected quantum kernel, where a small geometric difference *g* leads to a very good performance of classical ML models and no large quantum advantage can be seen. Only when the geometric difference *g* is large (projected kernel method with embedding E3) can we see some mild advantage over the best classical method. This result holds disregarding any detail of the quantum evolution we are trying to learn, even for ones that are hard to simulate classically.

In order to push the limits of separation between quantum and classical approaches in a learning setting, we now consider a set of engineered datasets with function values designed to saturate the geometric inequality ${s}_{{\rm{C}}}\le g{({K}^{{\rm{C}}}| | {K}^{{\rm{PQ}}})}^{2}{s}_{{\rm{PQ}}}$ between classical ML models with associated kernels and the projected quantum kernel method. In particular, we design the dataset such that *s*_PQ_ = 1 and ${s}_{{\rm{C}}}=g{({K}^{{\rm{C}}}| | {K}^{{\rm{PQ}}})}^{2}$. Recall from Eq. (3), this dataset will hence show the largest separation in the prediction error bound $\sqrt{s(N)/N}$. The engineered dataset is constructed via a simple eigenvalue problem with the exact procedure described in Supplementary Section 7 and the results are shown in Fig. 4. As the quantum nature of the encoding increases from E1 to E3, corresponding to increasing *g*, the performance of both the best classical methods and the original quantum kernel decline precipitously. The advantage of the projected quantum kernel closely follows the geometric difference *g* and reaches more than 20% for large sizes. Despite the optimization of *g* only being possible for classical methods with an associated kernel, the performance advantage remains stable across other common classical methods. Note that we also constructed engineered datasets saturating the geometric inequality between classical ML and the original quantum kernel, but the small geometric difference *g* presented no empirical advantage at large system size (see Supplementary Section 13).

In keeping with our arguments about the role of data, when we increase the number of training data *N*, all methods improve, and the advantage will gradually diminish. While this dataset is engineered, it shows the strongest empirical separation on the largest system size to date. We conjecture that this procedure could be used with a quantum computer to create challenging datasets that are easy to learn with a quantum device, hard to learn classically, while still being easy to verify classically given the correct labels. Moreover, the size of the margin implies that this separation may even persist under moderate amounts of noise in a quantum device.

## Discussion

The use of quantum computing in machine learning remains an exciting prospect, but quantifying quantum advantage for such applications has some subtle issues that one must approach carefully. Here, we constructed a foundation for understanding opportunities for quantum advantage in a learning setting. We showed quantitatively how classical ML algorithms with data can become computationally more powerful, and a prediction advantage for quantum models is not guaranteed even if the data come from a quantum process that is challenging to independently simulate. Motivated by these tests, we introduced projected quantum kernels. On engineered datasets, projected quantum kernels outperform all tested classical models in prediction error. To the authors’ knowledge, this is the first empirical demonstration of such a large separation between quantum and classical ML models.

This work suggests a simple guidebook for generating ML problems which give a large separation between quantum and classical models, even at a modest number of qubits. The size of this separation and trend up to 30 qubits suggests the existence of learning tasks that may be easy to verify, but hard to model classically, requiring just a modest number of qubits and allowing for device noise. Claims of true advantage in a quantum machine learning setting require not only benchmarking classical machine learning models, but also classical approximations of quantum models. Additional work will be needed to identify embeddings that satisfy the sometimes conflicting requirements of being hard to approximate classically and exhibiting meaningful signal on local observables for very large numbers of qubits. Further research will be required to find use cases on datasets closer to practical interest and evaluate potential claims of advantage, but we believe the tools developed in this work will help to pave the way for this exciting frontier.

## Supplementary information

Supplementary Information

## Data Availability

All other data that support the plots within this paper and other findings of this study are available upon reasonable request. Source data are provided with this paper.

## Source data

Source Data

## Code availability

A tutorial for reproducing smaller numerical experiments is available at https://www.tensorflow.org/quantum/tutorials/quantum_data.
