# Supplementary material for: Power of data in quantum machine learning
Source: Nat Commun. 2021 May 11;12:2631. doi: 10.1038/s41467-021-22539-9 (PMC8113501; doi:10.1038/s41467-021-22539-9)
Supplement: Supplementary file 1 — Supplementary Information [file 41467_2021_22539_MOESM1_ESM.pdf]

Power of data in quantum machine learning

Hsin-Yuan Huang,[1,2,3] Michael Broughton,[1] Masoud Mohseni,[1] Ryan Babbush,[1] Sergio Boixo,[1] Hartmut Neven,[1] and Jarrod R. McClean[1,*]

[1]*Google Research, 340 Main Street, Venice, CA 90291, USA*
[2]*Institute for Quantum Information and Matter, Caltech, Pasadena, CA, USA*
[3]*Department of Computing and Mathematical Sciences, Caltech, Pasadena, CA, USA*

Supplementary information

1. RIGOROUS PROOFS FOR STATEMENTS REGARDING THE MOTIVATING EXAMPLE

We first give a simple proof that the motivating example $f(\mathbf{x})$ considered in the main text is in general hard to compute classically. Then, we show that training a classical ML model to predict the function $f(\mathbf{x})$ is easy on a classical computer.

Proposition 1 (Restatement of Proposition 1). *Consider input vector $\mathbf{x} \in \mathbb{R}^p$ encoded into an n-qubit state $|\mathbf{x}\rangle = \sum_{k=1}^{p} x_k |k\rangle$. If a randomized classical algorithm can compute*

$$f(\mathbf{x}) = \langle \mathbf{x}| U_{QNN}^\dagger O U_{QNN} |\mathbf{x}\rangle \tag{S1}$$

up to 0.15-error with high probability over the randomness in the classical algorithm for any n, U_{QNN} and O in a time polynomial to the description length of U_{QNN} and O, the input vector size p, and the qubit system size n, then

$$BPP = BQP. \tag{S2}$$

Proof. We consider $p = 1$ and $|\mathbf{x}\rangle = |0^n\rangle$ the all zero computational basis state. A language L is in BQP if and only if there exists a polynomial-time uniform family of quantum circuits $\{Q_n : n \in \mathbb{N}\}$, such that

1. For all $n \in \mathbb{N}$, Q_n takes an n-qubit computational basis state as input, apply Q_n on the input state, and measures the first qubit in the computational basis as output.

2. For all $z \in L$, the probability that output of $Q_{|z|}$ applying on the input z is one is greater than or equal to 2/3.

3. For all $z \notin L$, the probability that output of $Q_{|z|}$ applying on the input z is zero is greater than or equal to 2/3.

If we have the randomized classical algorithm that can compute $f(x)$, then for all z: input bitstring, we consider the unitary quantum neural network given by

$$U_{\mathrm{QNN}} = Q_{|z|} \bigotimes_{i=1}^{n} X_i^{z_i}, \tag{S3}$$

where X_i is the Pauli-X matrix acting on the i-th qubit, and the observable O is given by Z_1. Hence, we have

1. For all $z \in L$, $f(\mathbf{x}) = \langle \mathbf{x}| U_{\mathrm{QNN}}^\dagger O U_{\mathrm{QNN}} |\mathbf{x}\rangle = \langle z| Q_{|z|}^\dagger Z_1 Q_{|z|} |z\rangle = \Pr[$the output of $Q_{|z|}$ applying on the input z is one $] - \Pr[$the probability that output of $Q_{|z|}$ applying on the input z is zero$] \geq 2/3 - 1/3 = 1/3$.

2. For all $z \notin L$, $f(\mathbf{x}) = \langle \mathbf{x}| U_{\mathrm{QNN}}^\dagger O U_{\mathrm{QNN}} |\mathbf{x}\rangle = \langle z| Q_{|z|}^\dagger Z_1 Q_{|z|} |z\rangle = \Pr[$the output of $Q_{|z|}$ applying on the input z is one $] - \Pr[$the probability that output of $Q_{|z|}$ applying on the input z is zero$] \leq 1/3 - 2/3 = -1/3$.

* Corresponding author: jmcclean@google.com

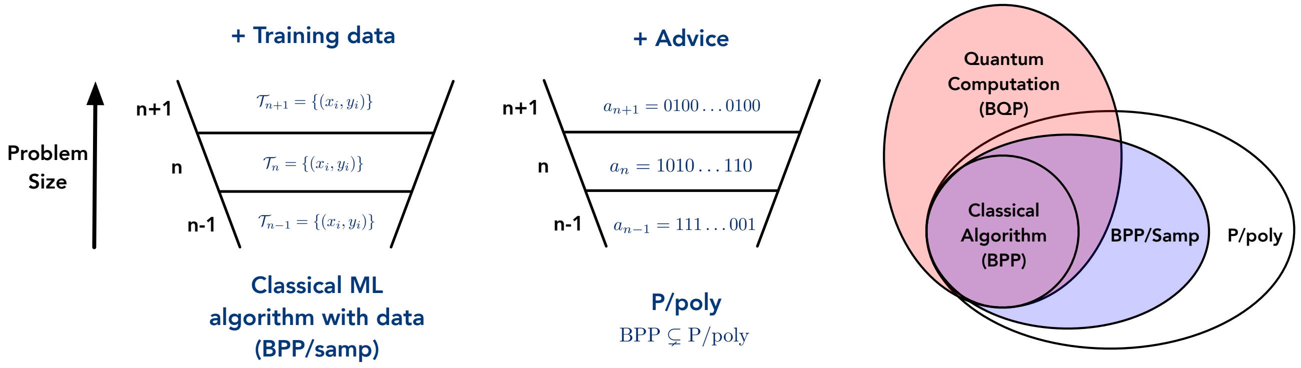

Supplementary Figure 1. We present an illustration of the complexity class for classical machine learning algorithm with the availability of data. To the right, we have a diagram showing the relations between different complexity classes.

By assumption, we can use the randomized classical algorithm to compute an estimate $\hat{f}(\mathbf{x})$ such that $|\hat{f}(\mathbf{x}) - f(\mathbf{x})| < 0.15$ with high probability over the randomness of the classical algorithm. Therefore with high probability, $\hat{f}(\mathbf{x}) > 0$ if $z \in L$ and $\hat{f}(\mathbf{x}) < 0$ if $z \notin L$. We can use the indication of whether $\hat{f}(\mathbf{x})$ is positive or negative to determine if $z \in L$ or $z \notin L$ with high probability over the randomness of the classical algorithm. This implies that $L \in \mathrm{BPP}$.

Together, the existence of the randomized classical algorithm implies that $\mathrm{BQP} \subseteq \mathrm{BPP}$. By definition, we have $\mathrm{BPP} \subseteq \mathrm{BQP}$, hence $\mathrm{BPP} = \mathrm{BQP}$.

We will now give a classical machine learning algorithm that could learn $f(\mathbf{x})$ efficiently using few samples. Recall that the data point is given by $\{\mathbf{x}_i\}_{i=1}^N$, where $\mathbf{x}_i \in \mathbb{R}^p$. Now, we consider a classical ML model with the kernel function $k(\mathbf{x}_i, \mathbf{x}_j) = (\sum_{l=1}^p x_{il} x_{jl})^2$, which can be evaluated in time linear in the dimension p. Note that this definition of kernel is equivalent to the quantum kernel $\mathrm{Tr}(\rho(x_i)\rho(x_j)) = |\langle x_i | x_j \rangle|^2$ for the encoding $|x_i\rangle = \sum_{k=1}^p x_{ik} |k\rangle$. We will now use the theoretical framework we developed in the main text (the section on testing quantum advantage). In particular, we will use the prediction error of quantum kernel method given in Eq. 8. It shows that for any observable O and quantum neural network U_{QNN}, the prediction error after training from N data points $\{(x_i, y_i = f(x_i))\}$ is given by

$$\mathbb{E}_{\mathbf{x} \in \mathcal{D}} |h(\mathbf{x}) - f(\mathbf{x})| \leq c \sqrt{\frac{\min(d, \mathrm{Tr}(O^2))}{N}}, \tag{S4}$$

where d is the Hilbert space dimension of $\{\rho(x_i)\}_{i=1}^N$. Because we have $\rho(x_i) = |x_i\rangle\langle x_i|$ and $|x_i\rangle = \sum_{k=1}^p x_{ik} |k\rangle$, the dimension of the Hilbert space is upper bounded by p^2. Therefore,

$$\mathbb{E}_{\mathbf{x} \in \mathcal{D}} |h(\mathbf{x}) - f(\mathbf{x})| \leq c \sqrt{\frac{\min(d, \mathrm{Tr}(O^2))}{N}} \leq c \sqrt{\frac{p^2}{N}}. \tag{S5}$$

This is the result stated in the main text. For more details about the machine learning models, the prediction error bound, and the proof for the prediction error bound of quantum kernel methods, see Supplementary Section 4 and 5 A.

2. COMPLEXITY-THEORETIC ARGUMENT FOR THE POWER OF DATA

In the main text, we give an argument based on an example to demonstrate the power of data. However, this is not satisfactory when we want to put the power of data on a rigorous footing. To demonstrate this fact from a rigorous standpoint, let us capture classical ML algorithms that can learn from data by means of a complexity class, which we refer to as BPP/samp. A language L of bit strings is in BPP/samp if and only if the following holds: There exists probabilistic Turing machines D and M. D generates samples x with $|x| = n$ in polynomial time for any input size n. D defines a sequence of input distributions $\{\mathcal{D}_n\}$. M takes an input x of size n along with $\mathcal{T} = \{(x_i, y_i)\}_{i=1}^{\mathrm{poly}(n)}$ of polynomial size, where x_i is sampled from $\mathcal{D}_n$ using Turing machine D and y_i conveys language membership: $y_i = 1$ if $x_i \in L$ and $y_i = 0$ if $x_i \notin L$. Moreover, we require

- The probabilistic Turing machine M to process all inputs x in polynomial time (polynomial runtime).
- For all $x \in L$, M outputs 1 with probability greater than or equal to 2/3 (prob. completeness).

- For all $x \notin L$, M outputs 1 with probability less than or equal to 1/3 (prob. soundness).

If the Turing machine M neglects the sampled data $\mathcal{T}$, this is equivalent to the definition of BPP. Hence BPP is contained inside BPP/samp.

We can also see that $\mathcal{T}$ is a restricted form of randomized advice string. It is not hard to show that BPP/samp is contained in P/poly based on the same proof strategy for Adleman's theorem. We consider a new probabilistic Turing machine M' that runs M for $18n$ times. Each time, we use an independently sampled training set $\mathcal{T}$ from $\mathcal{D}_n$. Then we take a majority vote from the $18n$ runs. By Chernoff bound, the probability of failure for any given x with $|x| = n$ would be at most $1/\mathrm{e}^n$. Hence by union bound, the probability that all x with $|x| = n$ succeeds is at least $1 - (2/\mathrm{e})^n$. This implies the existence of a particular choice of the $18n$ training sets and $18n$ random bit-strings used in each run of the probabilistic Turing machine M, such that for all x with $|x| = n$ the decision of whether $x \in L$ is correct. We simply define the advice string a_n to one particular choice of the $18n$ training sets and $18n$ random bit-strings, which will be a string of size polynomial in n. Hence we know that BPP/samp is contained in P/poly. An illustration is given in Supplementary Figure 1. We leave open the question of whether BPP/samp is strictly contained in P/poly.

The separation between P/poly and BPP is often illustrated by undecidable unary languages. The separation between BPP/samp and BPP could also be proved using a similar example. Actually, an undecidable unary language serves as an equally good example. Here, we choose to present a slightly more complicated example to demonstrate what BPP/samp could do. Let us consider an undecidable unary language $L_{\mathrm{hard}} = \{1^n | n \in A\}$, where A is a subset of the natural numbers $\mathbb{N}$ and a classically easy language $L_{\mathrm{easy}} \in$ BPP. We assume that for every input size n, there exists an input $a_n \in L_{\mathrm{easy}}$ and an input $b_n \notin L_{\mathrm{easy}}$. We define a new language as follows:

$$L = \bigcup_{n=1}^{\infty} \{x | \forall x \in L_{\mathrm{easy}}, 1^n \in L_{\mathrm{hard}}, |x| = n\} \cup \{x | \forall x \notin L_{\mathrm{easy}}, 1^n \notin L_{\mathrm{hard}}, |x| = n\}. \tag{S6}$$

For each size n, if $1^n \in L_{\mathrm{hard}}$, L would include all $x \in L_{\mathrm{easy}}$ with $|x| = n$. If $1^n \notin L_{\mathrm{hard}}$, L would include all $x \notin L_{\mathrm{easy}}$ with $|x| = n$. By definition, if we can output whether $x \in L$ for an input x using a classical algorithm (BPP), we can output whether $1^n \in L_{\mathrm{hard}}$ by computing whether $x \in L_{\mathrm{easy}}$. This is however impossible due to the undecidability of L_{hard}. Hence the language L is not in BPP. On the other hand, for every size n, a classical machine learning algorithm can use a single training data point (x_0, y_0) to decide whether $x \in L$. An algorithm is as follows. Using y_0, we know whether $x_0 \in L_{\mathrm{easy}}$. Hence, we know whether $1^n \in L_{\mathrm{hard}}$. Then for any input x with size n, we can output the correct answer by using the knowledge of whether $1^n \in L_{\mathrm{hard}}$ combined with a classical computation to decide whether $x \in L_{\mathrm{easy}}$. This example nicely illustrates the power of data and how machine learning algorithms can utilize it. In summary, the data provide information that is hard to compute with a classical computer (e.g., whether $1^n \in L_{\mathrm{hard}}$). Then the classical machine learning algorithm would perform classical computation to infer the solution from the given knowledge (e.g., computing whether $x \in L_{\mathrm{easy}}$). The same language L also yields a separation between BPP/samp and BQP because L is constructed to be undecidable.

From a practical perspective, it is impossible to obtain training data that is undecidable. But it is still possible to obtain data that cannot be efficiently computed with a classical computer, since the universe operates quantum mechanically. If the universe computes classically, then the data we can obtain will be computable by BPP and there is no separation between classical ML algorithm with data from BPP and BPP. We now present a simple argument for a separation between classical algorithm learning with data coming from quantum computation and BPP. This follows from a similar argument as the previous example. Here, we assume that there is a sequence of quantum circuits such that the Z measurement on the first qubit (being +1 with probability $> 2/3$ or $< 1/3$) is hard to decide classically. This defines a unary language L'_{hard} that is outside BPP, but inside BQP. We can then use L'_{hard} in replace of L_{hard} for the example above. When the data comes from BQP, the class classical ML algorithms that can learn from the data would not have a separation from BQP.

3. RELATION BETWEEN QUANTUM KERNEL METHODS AND QUANTUM NEURAL NETWORKS

In this section we demonstrate the formal equivalence of an arbitrary depth neural network with a quantum kernel method built from the original quadratic quantum kernel. This connection helps demonstrate the feature map induced by this kernel to motivate its use as opposed to the simpler inner product. While this equivalence shows the flexibility of this quantum kernel, it does not imply that it allows learning with a parsimonious amount of data. Indeed, in many cases it requires both an exponential amount of data and exponential precision in evaluation due to the fidelity type metric. In later sections we show simple cases where it fails for illustration purposes.

Proposition 2. *Training an arbitrarily deep quantum neural network U_{QNN} with a trainable observable O is equivalent to training a quantum kernel method with kernel $k_Q(x_i, x_j) = \mathrm{Tr}(\rho(x_i)\rho(x_j))$.*

Proof. Let us define $\rho_i = \rho(x_i) = U_{\text{enc}}(x_i) |0^n\rangle\langle 0^n| U_{\text{enc}}(x_i)^\dagger$ to be the corresponding quantum states for the classical input x_i. The training of a quantum neural network can be written‘ as

$$\min_{U \in \mathcal{C} \subset U(2^n)} \sum_{i=1}^{N} l(\text{Tr}(OU\rho_i U^\dagger), y_i), \tag{S7}$$

where $l(\tilde{y}, y)$ is a loss function that measures how close the prediction $\tilde{y}$ is to the true label y, $\mathcal{C}$ is the space of all possible unitaries considered by the parameterized quantum circuit, O is some predefined observable that we measure after evolving with U. Let us denote the optimal U to be U^*, then the prediction for a new input x is given by $\text{Tr}(OU^*\rho(x)(U^*)^\dagger)$.

On the other hand, the training of the quantum kernel method under the implied feature map is equivalent to training $W \in \mathbb{C}^{2^n \times 2^n}$ under the optimization

$$\min_{W \in \mathbb{C}^{2^n \times 2^n}} \sum_{i=1}^{N} l(\text{Tr}(W\rho_i), y_i) + \lambda \, \text{Tr}(W^\dagger W), \tag{S8}$$

where $\lambda \geq 0$ is the regularization parameter and $l(\tilde{y}, y)$ is the loss function. Let us denote the optimal W to be W^*, then the prediction for a new input x is given by $\text{Tr}(W^*\rho(x))$. The well-known kernel trick allows efficient implementation of this machine learning model, and connects the original quantum kernel to the derivation here. Using the fact that ρ_i is Hermitian and set $\lambda = 0$, the quantum kernel method can be expressed as

$$\min_{\substack{U \in U(2^n), \\ O \in \mathbb{C}^{2^n \times 2^n}, O = O^\dagger}} \sum_{i=1}^{N} l(\text{Tr}(OU\rho_i U^\dagger), y_i). \tag{S9}$$

This is equivalent to training an arbitrarily deep quantum neural network U with a trainable observable O.

4. PROOF OF A GENERAL FORM OF PREDICTION ERROR BOUND

This section is dedicated to deriving the precise statement for the core prediction error bound from which we base our methodology: $\mathbb{E}_x|h(x) - f(x)| \leq \mathcal{O}(\sqrt{s/N})$ given by the first inequality in Equation (3). We will provide a detailed proof for the following general theorem when we include the regularization parameter λ. The regularization parameter λ will be used to improve prediction performance by limiting the complexity of the machine learning model.

Theorem 1. *Consider an observable O with $\|O\|_\infty \leq 1$, a quantum unitary U (e.g., a quantum neural network or a general Hamiltonian evolution), a mapping of classical input x to quantum system $\rho(x)$, and a training set of N data $\{(x_i, y_i = \text{Tr}(O^U \rho(x_i)))\}_{i=1}^N$, with $O^U = U^\dagger O U$ being the Heisenberg evolved observable. The training set is sampled from some unknown distribution over the input x. Suppose that $k(x, x')$ can be evaluated efficiently and the kernel function is re-scaled to satisfy $\sum_{i=1}^N k(x_i, x_i) = N$. Define the Gram matrix $K_{ij} = k(x_i, x_j)$. For any $\lambda \geq 0$, with probability at least $1 - \delta$ over the sampling of the training data, we can learn a model $h(x)$ from the training data, such that the expected prediction error is bounded by*

$$\mathbb{E}_x|h(x) - \text{Tr}(O^U\rho(x))| \leq \mathcal{O}\left(\sqrt{\frac{\text{Tr}(A_{\text{tra}} O^U \otimes O^U)}{N}} + \sqrt{\frac{\text{Tr}(A_{\text{gen}} O^U \otimes O^U)}{N}} + \sqrt{\frac{\log(1/\delta)}{N}}\right), \tag{S10}$$

where the two operators $A_{\text{tra}}, A_{\text{gen}}$ are given as

$$A_{\text{tra}} = \lambda^2 \sum_{i=1}^{N} \sum_{j=1}^{N} ((K + \lambda I)^{-2})_{ij} \rho(x_i) \otimes \rho(x_j), \tag{S11}$$

$$A_{\text{gen}} = \sum_{i=1}^{N} \sum_{j=1}^{N} ((K + \lambda I)^{-1} K (K + \lambda I)^{-1})_{ij} \rho(x_i) \otimes \rho(x_j). \tag{S12}$$

This is a data-dependent bound as A_{tra} and A_{gen} both depend on the N training data.

When we take the limit of $\lambda \to 0$, we have $A_{\text{tra}} = 0$ and $A_{\text{gen}} = \sum_{i=1}^N \sum_{j=1}^N (K^{-1})_{ij} \rho(x_i) \otimes \rho(x_j)$. Thus with probability at least $0.99 = 1 - \delta$, we have

$$\mathbb{E}_x |h(x) - \text{Tr}(O^U \rho(x))| \leq \mathcal{O}\left(\sqrt{\frac{s_K(N)}{N}}\right), \tag{S13}$$

where $s_K(N) = \sum_{i=1}^N \sum_{j=1}^N (K^{-1})_{ij} \,\text{Tr}(O^U \rho(x_i))\, \text{Tr}(O^U \rho(x_j))$. This is the formula stated in the main text. However, in practice, we would recommend the use of regularization $\lambda > 0$ to prevent numerical instability and to obtain prediction error bound when we use a regularized ML model.

In Section 4 A, we will present the definition of the machine learning models used to prove Theorem 1. In Section 4 B and 4 C, we will analyze the training error and generalization error of the machine learning models we consider to prove the prediction error bound given in Theorem 1.

A. Definition and training of machine learning models

We consider a class of machine learning models, including Gaussian kernel regression, infinite-width neural networks, and quantum kernel methods. These models are equivalent to training a linear function mapping from a (possibly infinite-dimensional) Hilbert space $\mathcal{H}$ to $\mathbb{R}$. The linear function can be written as $\langle \mathbf{w}, \phi(x) \rangle$, where $\mathbf{w}$ parameterizes the linear function, $\langle \cdot, \cdot \rangle : \mathcal{H} \times \mathcal{H} \to \mathbb{R}$ is an inner product, and $\phi(x)$ is a nonlinear mapping from the classical input x to the Hilbert space $\mathcal{H}$. For example, in quantum kernel method, we use the space of $2^n \times 2^n$ Hermitian matrices as the Hilbert space $\mathcal{H}$. This yields a natural definition of inner product $\langle \rho, \sigma \rangle = \text{Tr}(\rho\sigma) \in \mathbb{R}$.

Because the output $y = \text{Tr}(U^\dagger O U \rho(x))$ of the quantum model satisfies $y \in [-1, 1]$, we confine the output of the machine learning model to the interval $[-1, 1]$. The resulting machine learning model would be

$$h_w(x) = \min(1, \max(-1, \langle \mathbf{w}, \phi(x) \rangle)). \tag{S14}$$

For efficient optimization of $\mathbf{w}$, we consider minimization of the following loss function

$$\min_{\mathbf{w}} \lambda \langle \mathbf{w}, \mathbf{w} \rangle + \sum_{i=1}^N \left(\langle \mathbf{w}, \phi(x) \rangle - \text{Tr}(U^\dagger O U \rho(x_i)) \right)^2, \tag{S15}$$

where $\lambda \geq 0$ is a hyper-parameter. We define $\Phi = (\phi(x_1), \ldots, \phi(x_N))$. The kernel matrix $K = \Phi^\dagger \Phi$ is an $N \times N$ matrix that defines the geometry between all pairs of the training data. We see that $K_{ij} = \langle \phi(x_i), \phi(x_j) \rangle = k(x_i, x_j) \in \mathbb{R}$. Without loss of generality, we consider $\text{Tr}(K) = N$, which can be done by rescaling $k(x_i, x_j)$. The optimal $\mathbf{w}$ can be written down explicitly as

$$\mathbf{w} = \sum_{i=1}^N \sum_{j=1}^N \phi(x_i) ((K + \lambda I)^{-1})_{ij} \,\text{Tr}(U^\dagger O U \rho(x_j)). \tag{S16}$$

Hence the trained machine learning model would be

$$h_w(x) = \min\left(1, \max\left(-1, \sum_{i=1}^N \sum_{j=1}^N k(x_i, x) ((K + \lambda I)^{-1})_{ij} \,\text{Tr}(U^\dagger O U \rho(x_j))\right)\right). \tag{S17}$$

This is an analytic representation for various trained machine learning models, including least-square support vector machine [1], kernel regression [2, 3], and infinite-width neural networks [4]. We will now analyze the prediction error of these machine learning models:

$$\epsilon_w(x) = |h_w(x) - \text{Tr}(U^\dagger O U \rho(x))|, \tag{S18}$$

which is uniquely determined by the kernel matrix K and the hyper-parameter λ. In particular, we will focus on providing an upper bound on the expected prediction error

$$\mathbb{E}_x \, \epsilon_w(x) = \underbrace{\frac{1}{N} \sum_{i=1}^N \epsilon_w(x_i)}_{\text{Training error}} + \underbrace{\mathbb{E}_x \, \epsilon_w(x) - \frac{1}{N} \sum_{i=1}^N \epsilon_w(x_i)}_{\text{Generalization error}}, \tag{S19}$$

which is the sum of training error and generalization error.

B. Training error

We will now relate the training error to the optimization problem, i.e., Equation (S15), for obtaining the machine learning model $h_w(x)$. Because $\|O\| \leq 1$, we have $\mathrm{Tr}(U^\dagger O U \rho(x)) \in [-1, 1]$, and hence $\epsilon_w(x) = |h_w(x) - \mathrm{Tr}(U^\dagger O U \rho(x))| \leq |\langle \mathbf{w}, \phi(x) \rangle - \mathrm{Tr}(U^\dagger O U \rho(x))|$. Using the convexity of x^2 and Jensen's inequality, we obtain

$$\frac{1}{N}\sum_{i=1}^{N} \epsilon_w(x_i) \leq \sqrt{\frac{1}{N}\sum_{i=1}^{N} \left(\langle \mathbf{w}, \phi(x)\rangle - \mathrm{Tr}(U^\dagger O U \rho(x_i))\right)^2}. \tag{S20}$$

We can plug in the expression for the optimal $\mathbf{w}$ given in Equation (S16) to yield

$$\frac{1}{N}\sum_{i=1}^{N} \epsilon_w(x_i) \leq \sqrt{\frac{\mathrm{Tr}(A_{\mathrm{tra}}(U^\dagger O U) \otimes (U^\dagger O U))}{N}}, \tag{S21}$$

where $A_{\mathrm{tra}} = \lambda^2 \sum_{i=1}^{N}\sum_{j=1}^{N} ((K + \lambda I)^{-2})_{ij} \rho(x_i) \otimes \rho(x_j)$. When K is invertible and $\lambda = 0$, we can see that the training error is zero. However, in practice, we often set $\lambda > 0$.

C. Generalization error

A basic theorem in statistics and learning theory is presented below. This theorem provides an upper bound on the largest (one-sided) deviation from expectation over a family of functions.

Theorem 2 (See Theorem 3.3 in [5]). *Let $\mathcal{G}$ be a family of function mappings from a set $\mathcal{Z}$ to $[0,1]$. Then for any $\delta > 0$, with probability at least $1 - \delta$ over identical and independent draw of N samples from $\mathcal{Z}$: $z_1, \ldots, z_N$, we have for all $g \in \mathcal{G}$,*

$$\mathbb{E}_z[g(z)] \leq \frac{1}{N}\sum_{i=1}^{N} g(z_i) + 2\mathbb{E}_\sigma\left[\sup_{g\in\mathcal{G}} \frac{1}{N}\sum_{i=1}^{N} \sigma_i g(z_i)\right] + 3\sqrt{\frac{\log(2/\delta)}{2N}}, \tag{S22}$$

where $\sigma_1, \ldots \sigma_N$ are independent and uniform random variables over ± 1.

For our purpose, we will consider $\mathcal{Z}$ to be the space of classical input with $z_i = x_i$ drawn from some input distribution. Each function g would be equal to $\epsilon_w/2$ for some $\mathbf{w}$, where ϵ_w is defined in Equation (S18). The reason that we divide by 2 is because the range of ϵ_w is $[0, 2]$. And $\forall \gamma = 1, 2, 3, \ldots$, we define $\mathcal{G}_\gamma$ to be $\{\epsilon_w/2 \mid \forall \|\mathbf{w}\| \leq \gamma\}$. The definition of an infinite sequence of family of functions $\mathcal{G}_\gamma$ is useful for proving a prediction error bound for an unbounded class of machine learning models $h_w(x)$, where $\|\mathbf{w}\|$ could be arbitrarily large. Using Theorem 2 and multiplying the entire inequality by 2, we can show that the following inequality holds for any $\mathbf{w}$ with $\|\mathbf{w}\| \leq \gamma$,

$$\mathbb{E}_x[\epsilon_w(x)] - \frac{1}{N}\sum_{i=1}^{N} \epsilon_w(x_i) \leq 2\mathbb{E}_\sigma\left[\sup_{\|\mathbf{v}\|\leq\gamma} \frac{1}{N}\sum_{i=1}^{N} \sigma_i \epsilon_{\mathbf{v}}(x_i)\right] + 6\sqrt{\frac{\log(4\gamma^2/\delta)}{2N}}, \tag{S23}$$

with probability at least $1 - \delta/2\gamma^2$. This probabilistic statement holds for any $\gamma = 1, 2, 3, \ldots$, but this does not yet guarantee that the inequality holds for all γ with high probability. We need to apply a union bound over all γ to achieve this, which shows that Inequality (S23) holds for all γ with probability at least $1 - \sum_{\gamma=1}^{\infty} \delta/2\gamma^2 \geq 1 - \delta$.

Together we have shown that, for any $\mathbf{w} \in \mathcal{H}$, the generalization error $\mathbb{E}_x[\epsilon_w(x)] - \frac{1}{N}\sum_{i=1}^{N} \epsilon_w(x_i)$ is upper bounded by

$$2\mathbb{E}_\sigma\left[\sup_{\|\mathbf{v}\|\leq\lceil\|\mathbf{w}\|\rceil} \frac{1}{N}\sum_{i=1}^{N} \sigma_i \epsilon_{\mathbf{v}}(x_i)\right] + 6\sqrt{\frac{\log(4\lceil\|\mathbf{w}\|\rceil^2/\delta)}{2N}}, \tag{S24}$$

with probability at least $1 - \delta$, where we consider the particular inequality with $\gamma = \lceil\|\mathbf{w}\|\rceil$. We will now analyze the above inequality using Talagrand's contraction lemma.

Lemma 1 (Talagrand's contraction lemma; See Lemma 5.7 in [5]). *Let $\mathcal{G}$ be a family of function from a set $\mathcal{Z}$ to $\mathbb{R}$. Let $l_1, \ldots, l_N$ be Lipschitz-continuous function from $\mathbb{R} \to \mathbb{R}$ with Lipschitz constant L. Then*

$$\mathbb{E}_\sigma\left[\sup_{g\in\mathcal{G}} \frac{1}{N}\sum_{i=1}^{N} \sigma_i l_i(g(z_i))\right] \leq L\mathbb{E}_\sigma\left[\sup_{g\in\mathcal{G}} \frac{1}{N}\sum_{i=1}^{N} \sigma_i g(z_i)\right]. \tag{S25}$$

We consider $l_i(s) = |\min(1, \max(-1, s)) - \mathrm{Tr}(U^\dagger O U \rho(x_i))|$, $z_i = x_i$, and $\mathcal{G} = \{g_v(z_i) = \langle \mathbf{v}, z_i \rangle \mid \|\mathbf{v}\| \leq \lceil \|\mathbf{w}\| \rceil\}$. This choice of functions gives $\epsilon_v(x_i) = l_i(g(z_i))$. Furthermore, l_i is Lipschitz-continuous with Lipschitz constant 1. Talagrand's contraction lemma then allows us to bound the formula in Equation (S24) by

$$2\mathbb{E}_\sigma \left[\sup_{\|\mathbf{v}\| \leq \lceil \|\mathbf{w}\| \rceil} \frac{1}{N} \sum_{i=1}^N \sigma_i \langle \mathbf{v}, \phi(x_i) \rangle \right] + 6\sqrt{\frac{\log(4\lceil \|\mathbf{w}\| \rceil^2/\delta)}{2N}} \tag{S26}$$

$$\leq 2\mathbb{E}_\sigma \left[\sup_{\|\mathbf{v}\| \leq \lceil \|\mathbf{w}\| \rceil} \frac{1}{N} \|\mathbf{v}\| \left\| \sum_{i=1}^N \sigma_i \phi(x_i) \right\| \right] + 6\sqrt{\frac{\log(4\lceil \|\mathbf{w}\| \rceil^2/\delta)}{2N}} \tag{S27}$$

$$\leq 2\lceil \|\mathbf{w}\| \rceil \mathbb{E}_\sigma \left[\frac{1}{N} \left\| \sum_{i=1}^N \sigma_i \phi(x_i) \right\| \right] + 6\sqrt{\frac{\log(4\lceil \|\mathbf{w}\| \rceil^2/\delta)}{2N}} \tag{S28}$$

$$\leq 2\frac{\lceil \|\mathbf{w}\| \rceil}{N} \sqrt{\mathbb{E}_\sigma \sum_{i=1}^N \sum_{j=1}^N \sigma_i \sigma_j k(x_i, x_j)} + 6\sqrt{\frac{\log(4\lceil \|\mathbf{w}\| \rceil^2/\delta)}{2N}} \tag{S29}$$

$$\leq 2\frac{\sqrt{\lceil \|\mathbf{w}\| \rceil^2 \mathrm{Tr}(K)}}{N} + 6\sqrt{\frac{\log(4\lceil \|\mathbf{w}\| \rceil^2/\delta)}{2N}} \tag{S30}$$

$$\leq 2\sqrt{\frac{\lceil \|\mathbf{w}\| \rceil^2}{N}} + 6\sqrt{\frac{\log(\lceil \|\mathbf{w}\| \rceil)}{N}} + 6\sqrt{\frac{\log(4/\delta)}{2N}} \tag{S31}$$

$$\leq 8\sqrt{\frac{\lceil \|\mathbf{w}\| \rceil^2}{N}} + 6\sqrt{\frac{\log(4/\delta)}{2N}}. \tag{S32}$$

The first inequality uses Cauchy's inequality. The second inequality uses the fact that $\|\mathbf{v}\| \leq \lceil \|\mathbf{w}\| \rceil$. The third inequality uses a Jensen's inequality to move $\mathbb{E}_\sigma$ into the square-root. The fourth inequality uses the fact that σ_i are independent and uniform random variable taking $+1, -1$. The fifth inequality uses $\sqrt{x+y} \leq \sqrt{x} + \sqrt{y}, \forall x, y \geq 0$ and our assumption that we rescale K such that $\mathrm{Tr}(K) = N$. The sixth inequality uses the fact that $x^2 \geq \log(x), \forall x \in \mathbb{N}$.

Finally, we plug in the optimal $\mathbf{w}$ given in Equation (S16). This allows us to obtain an upper bound of the generalization error:

$$\mathbb{E}_x[\epsilon_w(x)] - \frac{1}{N} \sum_{i=1}^N \epsilon_w(x_i) \leq 8 \frac{\lceil \sqrt{\mathrm{Tr}(A_{\mathrm{gen}}(U^\dagger O U) \otimes (U^\dagger O U))} \rceil}{\sqrt{N}} + 6\sqrt{\frac{\log(4/\delta)}{2N}}, \tag{S33}$$

where $A_{\mathrm{gen}} = \sum_{i=1}^N \sum_{j=1}^N ((K + \lambda I)^{-1} K (K + \lambda I)^{-1})_{ij} \rho(x_i) \otimes \rho(x_j)$. When K is invertible and $\lambda = 0$, we have $A_{\mathrm{gen}} = \sum_{i=1}^N \sum_{j=1}^N (K^{-1})_{ij} \rho(x_i) \otimes \rho(x_j)$.

5. SIMPLIFIED PREDICTION ERROR BOUND BASED ON DIMENSION AND GEOMETRIC DIFFERENCE

In this section, we will show that for quantum kernel methods, we have

$$\mathbb{E}_x |h^{\mathrm{Q}}(x) - \mathrm{Tr}(O^U \rho(x))| \leq \mathcal{O}\left(\sqrt{\frac{\min(d, \mathrm{Tr}(O^2))}{N}} \right), \tag{S34}$$

where d is the dimension of the training set space $d = \dim(\mathrm{span}(\rho(x_1), \ldots, \rho(x_N)))$. If we use the quantum kernel method as a reference point, then the prediction error of another machine learning algorithm that produces $h(x)$ using kernel matrix K can be bounded by

$$\mathbb{E}_x |h(x) - \mathrm{Tr}(O^U \rho(x))| \leq \mathcal{O}\left(g\sqrt{\frac{\min(d, \mathrm{Tr}(O^2))}{N}} \right), \tag{S35}$$

where $g = \sqrt{\left\| \sqrt{K^{\mathrm{Q}}} K^{-1} \sqrt{K^{\mathrm{Q}}} \right\|_\infty}$ assuming the normalization condition $\mathrm{Tr}(K^{\mathrm{Q}}) = \mathrm{Tr}(K) = N$.

A. Quantum kernel method

In quantum kernel method, the kernel function that will be used to train the model is defined using the quantum Hilbert space $k_Q(x, x') = \mathrm{Tr}(\rho(x)\rho(x'))$. Correspondingly, we define the kernel matrix $K^Q_{ij} = k_Q(x_i, x_j)$. We will focus on $\rho(x)$ being a pure state, so the scaling condition $\mathrm{Tr}(K^{\mathrm{Q}}) = \sum_{i=1}^N k_Q(x_i, x_i) = N$ is immediately satisfied. We also denote the trained model as h^{Q} for the quantum kernel method. We now consider an orthonormal basis $\{\sigma_1, \ldots, \sigma_d\}$ for the d-dimensional quantum state space formed by the training data $\mathrm{span}\{\rho(x_1), \ldots, \rho(x_N)\}$ under the inner product $\langle \rho, \sigma\rangle = \mathrm{Tr}(\rho\sigma)$. We have σ_p is Hermitian, $\mathrm{Tr}(\sigma_p^2) = 1$, but σ_p may not be positive semi-definite.

We consider an expansion of $\rho(x_i)$ in terms of σ_p:

$$\rho(x_i) = \sum_{p=1}^d \alpha_{ip}\sigma_p, \tag{S36}$$

where $\alpha \in \mathbb{R}^{N\times d}$. The coefficient α is real as the vector space of Hermitian matrices is over real numbers. Note that multiplying a Hermitian matrix with an imaginary number will not generally result in a Hermitian matrix, hence Hermitian matrices are not a vector space over complex numbers. We can perform a singular value decomposition on $\alpha = U\Sigma V^\dagger$, where $U \in \mathbb{C}^{N\times d}, \Sigma, V \in \mathbb{C}^{d\times d}$ with $U^\dagger U = I$, Σ is diagonal and $\Sigma \succ 0$, $V^\dagger V = VV^\dagger = I$. Then $K^{\mathrm{Q}} = \alpha\alpha^\dagger = U\Sigma^2U^\dagger$. This allows us to explicitly evaluate A_{tra} and A_{gen} given in Equation (S11) and (S12):

$$A_{\mathrm{tra}} = \lambda^2 \sum_{p=1}^d\sum_{q=1}^d \left(V\frac{\Sigma^2}{(\Sigma^2+\lambda I)^2}V^\dagger\right)_{pq} \sigma_p \otimes \sigma_q, \tag{S37}$$

$$A_{\mathrm{gen}} = \sum_{p=1}^d\sum_{q=1}^d \left(V\frac{\Sigma^4}{(\Sigma^2+\lambda I)^2}V^\dagger\right)_{pq} \sigma_p \otimes \sigma_q, \tag{S38}$$

which can be done by expanding $\rho(x_i)$ in terms of σ_p. Because $\Sigma \succ 0$, when we take the limit of $\lambda \to 0$, we have $A_{\mathrm{tra}} = 0$ and $A_{\mathrm{gen}} = \sum_{p=1}^d\sum_{q=1}^d \delta_{pq}\sigma_p\otimes\sigma_q = \sum_{p=1}^d \sigma_p\otimes\sigma_p$. Hence $\mathrm{Tr}(A_{\mathrm{tra}}O^U \otimes O^U) = 0$ and $\mathrm{Tr}(A_{\mathrm{gen}}O^U\otimes O^U) = \sum_{p=1}^d \mathrm{Tr}(\sigma_p O^U)^2$. From Equation (S10) with $\lambda \to 0$, we have

$$\mathbb{E}_x|h^{\mathrm{Q}}(x) - \mathrm{Tr}(O^U\rho(x))| \leq \mathcal{O}\left(\sqrt{\frac{\sum_{p=1}^d \mathrm{Tr}(O^U\sigma_p)^2}{N}} + \sqrt{\frac{\log(1/\delta)}{N}}\right). \tag{S39}$$

Because $\{\sigma_1, \ldots, \sigma_k\}$ forms an orthonormal set in the space of $2^n \times 2^n$ Hermitian matrices, $\sum_{p=1}^d \mathrm{Tr}(O^U\sigma_p)^2$ is the Frobenius norm of the observable O^U restricted to the subspace $\mathrm{span}\{\sigma_1, \ldots, \sigma_k\}$.

We now focus on obtaining an informative upper bound on how large $\sum_{p=1}^d \mathrm{Tr}(O^U\sigma_p)^2$ could be. First, because we can extend the subspace $\mathrm{span}\{\sigma_1, \ldots, \sigma_k\}$ to the full Hilbert space $\mathrm{span}\{\sigma_1, \ldots \sigma_{4^n}\}$, we have $\sum_{p=1}^d \mathrm{Tr}(O^U\sigma_p)^2 \leq \sum_{p=1}^{4^n} \mathrm{Tr}(O^U\sigma_p)^2 = \mathrm{Tr}((O^U)^2) = \left\|O^U\right\|_F^2$. Next, we will show that $\sum_{p=1}^d \mathrm{Tr}(O^U\sigma_p)^2 \leq d\left\|O^U\right\|_\infty^2 \leq d$, where $\left\|O^U\right\|_\infty$ is the spectral norm of the observable O^U. We pick a linearly-independent set of $\{\rho_1, \ldots, \rho_k\}$ from $\{\rho(x_1), \ldots \rho(x_N)\}$. We assume that all the quantum states are pure, hence we have $\rho_i = |\psi_i\rangle\langle\psi_i|, \forall i = 1, \ldots, d$. The pure states $\{|\psi_1\rangle, \ldots, |\psi_k\rangle\}$ may not be orthogonal, so we perform a Gram-Schmidt process to create an orthonormal set of quantum states $\{|\phi_1\rangle, \ldots, |\phi_k\rangle\}$. Because ρ_i are linear combination of $|\phi_q\rangle\langle\phi_r|, \forall q, r = 1, \ldots, d$, we have

$$\sigma_p = \sum_{q=1}^d\sum_{r=1}^d s_{pqr}\,|\phi_q\rangle\langle\phi_r|\,, \forall p = 1, \ldots, d. \tag{S40}$$

The condition $\mathrm{Tr}(\sigma_p\sigma_{p'}) = \delta_{pp'}$ implies that $\sum_{q=1}^d\sum_{r=1}^d s_{pqr}s_{p'qr} = \delta_{pp'}$. If we view s as a vector $\mathbf{s}$ of size d^2, then $\langle \mathbf{s}_p, \mathbf{s}_{p'}\rangle = \delta_{pp'}$. Thus $\{\mathbf{s}_1, \ldots, \mathbf{s}_k\}$ forms a set of orthonormal vectors in $\mathbb{R}^{d^2}$, which implies $\sum_{p=1}^d \mathbf{s}_p\mathbf{s}_p^\dagger \preceq I$. Let us define the projection operator $P = \sum_{q=1}^d |\phi_q\rangle\langle\phi_q|$. We will also consider a vector $\mathbf{o} \in \mathbb{R}^{d^2}$, where $\mathbf{o}_{qr} = \langle\phi_r|\,O^U\,|\phi_q\rangle$.

We have

$$\sum_{p=1}^{d} \mathrm{Tr}(O^U \sigma_p)^2 = \sum_{p=1}^{d} \left(\sum_{q=1}^{d} \sum_{r=1}^{d} s_{pqr} \langle \phi_r | O^U | \phi_q \rangle \right)^2 = \sum_{p=1}^{d} \mathbf{o}^\dagger \mathbf{s}_p \mathbf{s}_p^\dagger \mathbf{o} \tag{S41}$$

$$\leq \mathbf{o}^\dagger \mathbf{o} = \left(\sum_{q=1}^{d} \sum_{r=1}^{d} \langle \phi_r | O^U | \phi_q \rangle \right)^2 = \left\| P O^U P \right\|_F^2 . \tag{S42}$$

The inequality comes from the fact that $\sum_{p=1}^{d} \mathbf{s}_p \mathbf{s}_p^\dagger \preceq I$. With a proper choice of basis, one could view PO^UP as an $d \times d$ matrix. Hence $\left\|PO^UP\right\|_F \leq \sqrt{d} \left\|PO^UP\right\|_\infty \leq \sqrt{d} \left\|O^U\right\|_\infty$. This established the fact that $\sum_{p=1}^{d} \mathrm{Tr}(O^U\sigma_p)^2 \leq d \left\|O^U\right\|_\infty^2 \leq d$. Combining with Equation (S39), we have

$$\mathbb{E}_x |h^Q(x) - \mathrm{Tr}(O^U \rho(x))| \leq \mathcal{O}\left(\sqrt{\frac{\min(d, \|O^U\|_F^2)}{N}} + \sqrt{\frac{\log(1/\delta)}{N}} \right). \tag{S43}$$

This elucidates the fact that the prediction error of a quantum kernel method is bounded by minimum of the dimension of the quantum subspace formed by the training set and the Frobenius norm of the observable O^U.

Choosing a small but non-zero λ allows us to consider an approximate space of $\mathrm{span}\{\rho(x_1), \ldots, \rho(x_N)\}$ formed by the training set. The training error $\sqrt{\mathrm{Tr}(A_{\mathrm{tra}} O^U \otimes O^U)/N}$ would increase slightly, and the generalization error $\sqrt{\mathrm{Tr}(A_{\mathrm{gen}} O^U \otimes O^U)/N}$ would reflect the Frobenius norm of O^U restricted to a smaller subspace, which only contains the principal components of the space formed by the training set. This would be a better choice when most states lie in low-dimensional subspace with small random fluctuations. One may also consider training a machine learning model with truncated kernel matrix K_λ, where all singular values below λ are truncated. This makes the act of restricting to an approximate subspace more explicit.

B. Another machine learning method compared to quantum kernel

We now consider an upper bound on the prediction error using the quantum kernel method as a reference point for some machine learning algorithm. For the following discussion, we consider classical neural networks with large hidden sizes. The function generated by a classical neural network with large hidden size after training is equivalent to the function $h(x)$ given in Equation (S16) with $\lambda = 0$ and with a special kernel function $k_{\mathrm{NTK}}(x, x')$ known as the neural tangent kernel (NTK) [4]. The precise definition of $k_{\mathrm{NTK}}(x, x')$ depends on the architecture of the neural network. For example, a two-layer feedforward neural network (FNN), a three-layer FNN, or some particular form of convolutional neural network (CNN) all correspond to different $k_{\mathrm{NTK}}(x, x')$. Given the kernel $k_{\mathrm{NTK}}(x, x')$, we can define the kernel matrix $\tilde{K}_{ij} = k_{\mathrm{NTK}}(x_i, x_j)$. For neural tangent kernel, the scaling condition $\mathrm{Tr}(\tilde{K}) = \sum_{i=1}^{N} k_{\mathrm{NTK}}(x_i, x_i) = N$ may not be satisfied. Hence, we define a normalized kernel matrix $K = N\tilde{K}/\mathrm{Tr}(\tilde{K})$. When $\lambda = 0$, the trained machine learning model (given in Equation (S16)) under the normalized matrix K and the original matrix $\tilde{K}$ are the same. In order to apply Theorem 1, we will use the normalized kernel matrix K for the following discussion. From Equation (S10) with $\lambda = 0$, we have

$$\mathbb{E}_x |h(x) - \mathrm{Tr}(O^U \rho(x))| \leq \mathcal{O}\left(\sqrt{\frac{\mathrm{Tr}(A O^U \otimes O^U)}{N}} + \sqrt{\frac{\log(1/\delta)}{N}} \right), \tag{S44}$$

where $A = \sum_{i=1}^{N} \sum_{j=1}^{N} (K^{-1})_{ij} \rho(x_i) \otimes \rho(x_j)$. Using Equation (S36) on the expansion of $\rho(x_i)$, we have

$$A = \sum_{p=1}^{d} \sum_{q=1}^{d} \sum_{i=1}^{N} \sum_{j=1}^{N} (K^{-1})_{ij} \alpha_{ip} \alpha_{jq} \sigma_p \otimes \sigma_q \tag{S45}$$

$$= \sum_{p=1}^{d} \sum_{q=1}^{d} (\alpha^\dagger K^{-1} \alpha)_{pq} \sigma_p \otimes \sigma_q. \tag{S46}$$

Using the definition of spectral norm, we have

$$\operatorname{Tr}(AO^U \otimes O^U) = \sum_{p=1}^{d}\sum_{q=1}^{d}(\alpha^\dagger K^{-1}\alpha)_{pq} \operatorname{Tr}(\sigma_p O^U) \operatorname{Tr}(\sigma_q O^U) \tag{S47}$$

$$\leq \left\|\alpha^\dagger K^{-1}\alpha\right\|_\infty \sum_{p=1}^{d} \operatorname{Tr}(O^U\sigma_p)^2. \tag{S48}$$

Recall from the definition below Equation (S36), we have

$$\alpha = U\Sigma V^\dagger, K^{\mathrm{Q}} = \alpha\alpha^\dagger = U\Sigma^2 U^\dagger. \tag{S49}$$

Using the fact that orthogonal transformation do not change the spectral norm, $\left\|\alpha^\dagger K^{-1}\alpha\right\|_\infty = \left\|\Sigma U^\dagger K^{-1} U\Sigma\right\|_\infty = \left\|U\Sigma U^\dagger K^{-1} U\Sigma U^\dagger\right\|_\infty = \left\|\sqrt{K^{\mathrm{Q}}}K^{-1}\sqrt{K^{\mathrm{Q}}}\right\|_\infty$. Hence

$$\operatorname{Tr}(AO^U \otimes O^U) \leq \left\|\sqrt{K^{\mathrm{Q}}}K^{-1}\sqrt{K^{\mathrm{Q}}}\right\|_\infty \sum_{p=1}^{d} \operatorname{Tr}(O^U\sigma_p)^2. \tag{S50}$$

Together with Equation (S44), we have the following prediction error bound

$$\mathbb{E}_x|h(x) - \operatorname{Tr}(O^U\rho(x))| \leq \mathcal{O}\left(g\sqrt{\frac{\sum_{p=1}^d \operatorname{Tr}(O^U\sigma_p)^2}{N}} + \sqrt{\frac{\log(1/\delta)}{N}}\right), \tag{S51}$$

where $g = \sqrt{\left\|\sqrt{K^{\mathrm{Q}}}K^{-1}\sqrt{K^{\mathrm{Q}}}\right\|_\infty}$. The scalar g measures the closeness of the geometry between the training data points defined by classical neural network and quantum state space. Note that without the geometric scalar g, this prediction error bound is the same as Equation (S39) for the quantum kernel method. Hence, if g is small, classical neural network could predict as well (or potentially better) as the quantum kernel method. The same analysis in Section 5 A allows us to arrive at the following result

$$\mathbb{E}_x|h(x) - \operatorname{Tr}(O^U\rho(x))| \leq \mathcal{O}\left(g\sqrt{\frac{\min(d, \|O^U\|_F^2)}{N}} + \sqrt{\frac{\log(1/\delta)}{N}}\right). \tag{S52}$$

The same analysis holds for other machine learning algorithms, such as Gaussian kernel regression.

6. DETAILED DISCUSSION ON THE RELEVANT QUANTITIES S, D, AND G

There are some important aspects on the three relevant quantities s, d, g that were not fully discussed in the main text, including the limit when we have infinite amount of data and the effect of regularization. While in practice one always has a finite amount of data, constructing these formal limits both clarifies the construction and provides another perspective through which to understand the finite data constructions. This section will provide a detailed discussion of these aspects.

A. Model complexity s

While we have used $s_K(N) = \sum_{i=1}^N \sum_{j=1}^N (K^{-1})_{ij} \operatorname{Tr}(O^U\rho(x_i)) \operatorname{Tr}(O^U\rho(x_j))$ in the main text, this is a simplified quantity when we do not apply regularization. The model complexity $s_K(N)$ under regularization is given by

$$s_K(N) = \|\mathbf{w}\|^2 = \operatorname{Tr}(A_{\mathrm{gen}}O^U \otimes O^U) = \sum_{i=1}^{N}\sum_{j=1}^{N}((K+\lambda I)^{-1}K(K+\lambda I)^{-1})_{ij} \operatorname{Tr}(O^U\rho(x_i)) \operatorname{Tr}(O^U\rho(x_j)) \tag{S53}$$

$$= \sum_{i=1}^{N}\sum_{j=1}^{N}(\sqrt{K}(K+\lambda I)^{-2}\sqrt{K})_{ij} \operatorname{Tr}(O^U\rho(x_i)) \operatorname{Tr}(O^U\rho(x_j)). \tag{S54}$$

Training machine learning model with regularization is often desired when we have a finite number N of training data. $\|\mathbf{w}\|^2$ has been used extensively in regularizing machine learning models, see e.g., [1, 6, 7]. This is because we can often significantly reduce generalization error $\sqrt{\mathrm{Tr}(A_{\mathrm{gen}} O^U \otimes O^U)/N}$ by slightly increasing the training error $\sqrt{\mathrm{Tr}(A_{\mathrm{tra}} O^U \otimes O^U)/N}$. In practice, we should choose the regularization parameter λ to be a small number such that the training error plus the generalization error is minimized.

The model complexity $s_K(N)$ we have been calculating can be seen as an approximation to the true model complexity when we have a finite number N of training data. If we have exact knowledge about the input distribution given as a probability measure μ_x, we can also write down the precise model complexity in the reproducing kernel Hilbert space $\phi(x)$ where $k(x, y) = \phi(x)^\dagger \phi(y)$. Starting from

$$\min_{\mathbf{w}} \lambda \mathbf{w}^\dagger \mathbf{w} + \int \left\| \mathbf{w}^\dagger \phi(x) - \mathrm{Tr}(O^U \rho(x)) \right\|^2 d\mu_x, \tag{S55}$$

we can obtain

$$\mathbf{w} = \left(\lambda I + \int \phi(x)\phi(x)^\dagger d\mu_x \right)^{-1} \int \mathrm{Tr}(O^U \rho(x)) \phi(x) d\mu_x. \tag{S56}$$

Hence the true model complexity is

$$\|\mathbf{w}\|^2 = \int\int d\mu_{x_1} d\mu_{x_2} \, \mathrm{Tr}(O^U \rho(x_1)) \, \mathrm{Tr}(O^U \rho(x_2)) \phi(x_1)^\dagger \left(\lambda I + \int \phi(\xi)\phi(\xi)^\dagger d\mu_\xi \right)^{-2} \phi(x_2) \tag{S57}$$

$$= \mathrm{Tr}(A_{\mathrm{gen}} O^U \otimes O^U), \tag{S58}$$

where the operator

$$A_{\mathrm{gen}} = \int\int d\mu_{x_1} d\mu_{x_2} \phi(x_1)^\dagger \left(\lambda I + \int \phi(\xi)\phi(\xi)^\dagger d\mu_\xi \right)^{-2} \phi(x_2) \; \rho(x_1) \otimes \rho(x_2). \tag{S59}$$

If we replace the integration over the probability measure with N random samples and apply the fact that $k(x, y) = \phi(x)^\dagger \phi(y)$, then we can obtain the original expression given in Equation (S12).

B. Dimension d

The dimension we considered in the main text is the effective dimension of the training set quantum state space. This can be seen as the rank of the quantum kernel matrix $K^Q_{ij} = \mathrm{Tr}(\rho(x_i)\rho(x_j))$. However, it will often be the case that most of the states lie in some low-dimensional subspace, but have negligible contributions in a much higher dimensional subspace. In this case, the dimension of the low-dimensional subspace is the better characterization. More generally, we can perform a singular value decomposition of K^{Q}

$$K^{\mathrm{Q}} = \sum_{i=1}^{N} t_i u_i u_i^\dagger, \tag{S60}$$

with $t_1 \geq t_2 \geq \ldots \geq t_N$. We define $\sigma_i = \sum_{j=1}^N u_{ij} \rho(x_j) / \left\| \sum_{j=1}^N u_{ij} \rho(x_j) \right\|_F$, where $\|\cdot\|_F$ is the Frobenius norm. σ_i is the i-th principal component of the quantum state space. Recall the normalization condition $\mathrm{Tr}(K^{\mathrm{Q}}) = N$, so $\sum_{i=1}^N t_i = N$. If the training set quantum state space is one-dimensional ($d = 1$), then

$$t_1 = N, t_i = 0, \forall i > 1. \tag{S61}$$

If all the quantum states in the training set are orthogonal ($d = N$), then

$$t_i = 1, \forall i = 1, \ldots, N. \tag{S62}$$

By the Eckart-Young-Mirsky theorem, for any $k \geq 1$, the first k principal components $\sigma_1, \ldots, \sigma_k$ form the best k-dimensional subspace for approximating $\mathrm{span}\{\rho(x_1), \ldots, \rho(x_N)\}$. The approximation error is given by

$$\sum_{i=1}^{N} \left\| \rho(x_i) - \sum_{j=1}^{k} \sqrt{t_j} u_{ji} \sigma_j \right\|_F^2 = \sum_{l=k+1}^{N} t_l. \tag{S63}$$

As we can see, when the spectrum is flatter, the dimension is larger. The error decreases at most as $\sum_{l=k}^{N} t_l \leq N-k$, where the equality holds when all states are orthogonal. In the numerical experiment, we choose the following measure as the approximate dimension

$$1 \leq \sum_{k=1}^{N} \left(\frac{1}{N-k} \sum_{l=k}^{N} t_l \right) \leq N \tag{S64}$$

due to the independence to any hyperparameter. Alternatively, we can also define approximate dimension by choosing the smallest k such that $\sum_{l=k+1}^{N} t_l/N < \epsilon$ for some $\epsilon > 0$. Both give similar trend, but the actual value of the dimension would be different.

From the discussion, we can see that in the above definitions, the dimension will always be upper bounded by the number N of training data. Similar to the case of model complexity, we can also define the dimension d when we have the exact knowledge about the input distribution given by probability measure μ_x. For a quantum state space representing n qubits, we simply consider the spectrum $t_1 \geq t_2 \geq \ldots \geq t_{2^n}$ of the following operator

$$\int \mathrm{vec}(\rho(x)) \mathrm{vec}(\rho(x))^T d\mu_x. \tag{S65}$$

When we replace the integration by a finite number of training samples, the spectrum would be equivalent to the spectrum given in Equation (S60) except for the additional zeros.

Remark 1. *The same definition of dimension can be used for any kernels, such as projected quantum kernels or neural tangent kernels (under the normalization* $\mathrm{Tr}(K) = N$*).*

C. Geometric difference g

The geometric difference is defined between two kernel functions K^1, K^2 and the corresponding reproducing kernel Hilbert space $\phi_1(x), \phi_2(x)$. If we have a function represented by the first kernel $\mathbf{w}^\dagger \phi_1(x)$, what would be the model complexity for the second kernel? We consider the ideal case where we know the input distribution μ_x exactly. The optimization for training the first kernel method with regularization $\lambda > 0$ is

$$\min_{\mathbf{v}} \lambda \mathbf{v}^\dagger \mathbf{v} + \int \left\| \mathbf{v}^\dagger \phi_2(x) - \mathbf{w}^\dagger \phi_1(x) \right\|^2 d\mu_x. \tag{S66}$$

The solution is given by

$$\mathbf{v} = \left(\lambda I + \int \phi_2(x) \phi_2(x)^\dagger d\mu_x \right)^{-1} \int \mathbf{w}^\dagger \phi_1(x) \phi_2(x) d\mu_x. \tag{S67}$$

Hence the model complexity for the optimized $\mathbf{v}$ is

$$\|\mathbf{v}\|^2 = \mathbf{w}^\dagger \left(\int \int d\mu_{x_1} d\mu_{x_2} \phi_1(x_1) \phi_2(x_1)^\dagger \left(\lambda I + \int \phi_2(\xi) \phi_2(\xi)^\dagger d\mu_\xi \right)^{-2} \phi_2(x_2) \phi_1(x_2)^\dagger \right) \mathbf{w} \tag{S68}$$

$$\leq g_{\mathrm{gen}}^2 \|\mathbf{w}\|^2, \tag{S69}$$

where the geometric difference is

$$g_{\mathrm{gen}} = \sqrt{\left\| \int \int d\mu_{x_1} d\mu_{x_2} \phi_1(x_1) \phi_2(x_1)^\dagger \left(\lambda I + \int \phi_2(\xi) \phi_2(\xi)^\dagger d\mu_\xi \right)^{-2} \phi_2(x_2) \phi_1(x_2)^\dagger \right\|_\infty}. \tag{S70}$$

The subscript in g_{gen} is added because when $\lambda > 0$, there will also be a contribution from training error. When we only have a finite number N of training samples, we can use the fact that $k(x, y) = \phi(x)^\dagger \phi(y)$ and the definition that $K_{ij} = k(x_i, x_j)$ to obtain

$$g_{\mathrm{gen}} = \sqrt{\left\| \sqrt{K^1} \sqrt{K^2} \left(K^2 + \lambda I \right)^{-2} \sqrt{K^2} \sqrt{K^1} \right\|_\infty}. \tag{S71}$$

This formula differs from the main text due to the regularization parameter λ. If $\lambda = 0$, then the above formula for g_{gen} reduces to the formula $g_{\mathrm{gen}} = \sqrt{\left\|\sqrt{K^1}(K^2)^{-1}\sqrt{K^1}\right\|_\infty}$.

When λ is non-zero, the geometric difference can become much smaller. This is the same as the discussion on model complexity s in Section 6 A. However, a nonzero λ induces a small amount of training error. For a finite number N of samples, the training error can always be upper bounded:

$$\frac{1}{N}\sum_{i=1}^{N}\left\|\mathbf{v}^\dagger\phi_2(x_i) - \mathbf{w}^\dagger\phi_1(x_i)\right\|^2 \leq \lambda^2\left\|\sqrt{K^1}(K^2+\lambda I)^{-2}\sqrt{K^1}\right\|_\infty \|\mathbf{w}\|^2 = g_{\mathrm{tra}}^2\|\mathbf{w}\|^2, \tag{S72}$$

where $g_{\mathrm{tra}} = \lambda\sqrt{\left\|\sqrt{K^1}(K^2+\lambda I)^{-2}\sqrt{K^1}\right\|_\infty}$. This upper bound can be obtained by plugging the solution for $\mathbf{v}$ in Equation (S66) under finite samples into the training error $\frac{1}{N}\sum_{i=1}^{N}\left\|\mathbf{v}^\dagger\phi_2(x_i) - \mathbf{w}^\dagger\phi_1(x_i)\right\|^2$ and utilizing the fact that $\mathbf{w}^\dagger A\mathbf{w} \leq \|A\|_\infty\|\mathbf{w}\|^2$. In the numerical experiment, we report g_{gen} given in Equation (S71) with the largest λ such that the training error $g_{\mathrm{tra}} \leq 0.045$.

7. CONSTRUCTING DATASET TO SEPARATE QUANTUM AND CLASSICAL MODEL

In the main text, our central quantity of interest is the geometric difference g, which provides a quantification for a given data set, how large the prediction gap can be for possibly function or labels associated with that data. Here we detail how one can efficiently construct a function that saturates this bound for a given data set. This is the approach that is used in the main text to engineer the data set with maximal performance.

Given a (projected) quantum kernel $k^{\mathrm{Q}}(x_i,x_j) = \phi^{\mathrm{Q}}(x_i)^\dagger\phi^{\mathrm{Q}}(x_j)$ and a classical kernel $k^{\mathrm{C}}(x_i,x_j) = \phi^{\mathrm{C}}(x_i)^\dagger\phi^{\mathrm{C}}(x_j)$, our goal is to construct a dataset that would best separate the two models. Consider a dataset $(x_i, y_i), \forall i = 1,\ldots,N$. We use the model complexity $s = \sum_{i=1}^{N}\sum_{j=1}^{N}(K^{-1})_{ij}y_iy_j$ to quantify the generalization error of the model. The model complexity has been introduced in the main text, where a detailed proof relating s to prediction error is given in Supplementary Section 4. To separate between quantum and classical model, we consider $s_{\mathrm{Q}} = 1$ and s_{C} is as large as possible for a particular choice of targets $y_1,\ldots,y_N$. To achieve this, we solve the optimization

$$\min_{y\in\mathbb{R}^N}\frac{\sum_{i=1}^{N}\sum_{j=1}^{N}((K^{\mathrm{C}})^{-1})_{ij}y_iy_j}{\sum_{i=1}^{N}\sum_{j=1}^{N}((K^{\mathrm{Q}})^{-1})_{ij}y_iy_j} \tag{S73}$$

which has an exact solution given by a generalized eigenvalue problem. The solution is given by $y = \sqrt{K^{\mathrm{Q}}}\mathbf{v}$, where $\mathbf{v}$ is the eigenvector of $\sqrt{K^{\mathrm{Q}}}(K^{\mathrm{C}})^{-1}\sqrt{K^{\mathrm{Q}}}$ corresponding to the eigenvalue $g^2 = \left\|\sqrt{K^{\mathrm{Q}}}(K^{\mathrm{C}})^{-1}\sqrt{K^{\mathrm{Q}}}\right\|_\infty$. This guarantees that $s_{\mathrm{C}} = g^2 s_{\mathrm{Q}} = g^2$, and note that by definition of g, $s_{\mathrm{C}} \leq g^2 s_{\mathrm{Q}}$. Hence this dataset fully utilized the geometric difference between the quantum and classical space.

We should also include regularization parameter λ when constructing the dataset. Detailed discussion on model complexity s and geometric difference g with regularization is given in Supplementary Section 6. Recall that for $\lambda > 0$,

$$s_C^\lambda = y^\dagger(\sqrt{K^{\mathrm{C}}}\left(K^{\mathrm{C}}+\lambda I\right)^{-2}\sqrt{K^{\mathrm{C}}})_{ij}y, \tag{S74}$$

which is the model complexity that we want to maximize. Similar to the unregularized case, we consider the (unregularized) model complexity $s_{\mathrm{Q}} = y^\dagger(K^{\mathrm{Q}})^{-1}y$ to be one. Solving the generalized eigenvector problem yields the target $y = \sqrt{K^{\mathrm{Q}}}\mathbf{v}$, where $\mathbf{v}$ is the eigenvector of

$$\sqrt{K^{\mathrm{Q}}}\sqrt{K^{\mathrm{C}}}\left(K^{\mathrm{C}}+\lambda I\right)^{-2}\sqrt{K^{\mathrm{C}}}\sqrt{K^{\mathrm{Q}}} \tag{S75}$$

with the corresponding eigenvalue

$$g_{\mathrm{gen}}^2 = \left\|\sqrt{K^{\mathrm{Q}}}\sqrt{K^{\mathrm{C}}}\left(K^{\mathrm{C}}+\lambda I\right)^{-2}\sqrt{K^{\mathrm{C}}}\sqrt{K^{\mathrm{Q}}}\right\|_\infty. \tag{S76}$$

The larger λ is, the smaller g_{gen}^2 would be. In practice, one should choose a λ such that the training error bound $g_{\mathrm{tra}}^2 s_{\mathrm{Q}} = \lambda^2\left\|\sqrt{K^{\mathrm{Q}}}(K^{\mathrm{C}}+\lambda I)^{-2}\sqrt{K^{\mathrm{Q}}}\right\|_\infty$ for the classical ML model is small enough. In the numerical experiment, we choose a λ such that the training error bound $g_{\mathrm{tra}}^2 s_{\mathrm{Q}} \leq 0.002$ and g_{gen} is as large as possible. Finally, we can turn this

dataset, which maps input x to a real value y, into a classification task by replacing y with $+1$ if $y > \text{median}(y_1, \ldots, y_N)$ and -1 if $y \leq \text{median}(y_1, \ldots, y_N)$.

The constructed dataset will yield the largest separation between quantum and classical models from a learning-theoretic sense, as the model complexity fully saturates the geometric difference. If there is no quantum advantage in this dataset, there will likely be none. We believe this construction procedure will eventually lead to the first quantum advantage in machine learning problems (classification problems to be more specific).

8. LOWER BOUND ON LEARNING QUANTUM MODELS

In this section, we will prove a fundamental lower bound for learning quantum models stated in Theorem 3. This result says that in the worst case, the number N of training data has to be at least $\Omega(\text{Tr}(O^2)/\epsilon^2)$ when the input quantum state can be distributed across a sufficiently large Hilbert space. Quantum kernel method matches this lower bound. When the data spans over the entire Hilbert space, the dimension d will be large and the prediction error of the quantum kernel method given in Equation (S34) becomes

$$\mathbb{E}_x |h^{\text{Q}}(x) - \text{Tr}(O^U \rho(x))| \leq \mathcal{O}\left(\sqrt{\frac{\text{Tr}(O^2)}{N}}\right). \tag{S77}$$

Hence we can achieve ϵ error using $N \leq \mathcal{O}(\text{Tr}(O^2)/\epsilon^2)$ matching the fundamental lower bound.

Theorem 3. *Consider any learning algorithm $\mathcal{A}$. Suppose for any unknown unitary evolution U, any unknown observable O with bounded Frobenius norm $\text{Tr}(O^2) \leq B$, and any distribution D over the input quantum states, the learning algorithm $\mathcal{A}$ could learn a function h such that*

$$\mathbb{E}_{\rho \sim D} |h(\rho) - \text{Tr}(OU\rho U^\dagger)| \leq \epsilon, \tag{S78}$$

from N training data $(\rho_i, \text{Tr}(OU\rho_i U^\dagger)), \forall i = 1, \ldots, N$ with high probability. Then we must have

$$N \geq \Omega(B/\epsilon^2). \tag{S79}$$

Proof. We select a Hilbert space with dimension $d = B/4\epsilon^2$ (this could be a subspace of an exponentially large Hilbert space). We define the distribution D to be the uniform distribution over the basis states $|x\rangle\langle x|$ of the d-dimensional Hilbert space. Then we consider the unknown unitary U to always be the identity, while the possible observables are

$$O_v = 2\epsilon \sum_{x=1}^{d} v_x |x\rangle\langle x|, \tag{S80}$$

with $v_x \in \{\pm 1\}, \forall x = 1, \ldots, d$. There are hence 2^d different choices of observables O_v.

We now set up a simple communication protocol to prove the lower bound on the number of data needed. This is a simplified version of the proofs found in Refs.[8, 9]. Alice samples an observable O_v uniformly at random from the 2^d possible choices. We can treat v as a bit-string of d entries. Then she samples N quantum states $|x_i\rangle\langle x_i|, \forall i = 1, \ldots, N$. Alice then gives Bob the following training data $\mathcal{T} = \{(|x_i\rangle\langle x_i|, \langle x_i| O_v |x_i\rangle) = v_{x_i}, \forall i = 1, \ldots, N\}$. Notice that the mutual information $I(v, \mathcal{T})$ between v and the training data $\mathcal{T}$ satisfies

$$I(s, \mathcal{T}) \leq N, \tag{S81}$$

because the training data contains at most N values of s.

With high probability, the following is true by the requirement of the learning algorithm $\mathcal{A}$. Using the training data $\mathcal{T}$, Bob can apply the learning algorithm $\mathcal{A}$ to obtain a function f such that

$$\mathbb{E}_{\rho \sim D} |h(\rho) - \text{Tr}(OU\rho U^\dagger)| \leq \epsilon. \tag{S82}$$

Using Markov's inequality, we have

$$\Pr[|h(\rho) - \text{Tr}(OU\rho U^\dagger)| < 2\epsilon] > \frac{1}{2}. \tag{S83}$$

For all $x = 1, \ldots, d$, if $|h(|x\rangle\langle x|) - \text{Tr}(OU |x\rangle\langle x| U^\dagger)| < 2\epsilon$, we have $|h(|x\rangle\langle x|)/2\epsilon - v_x| < 1$. This means that if $h(|x\rangle\langle x|) > 0$, then $v_x = 1$ and if $h(|x\rangle\langle x|) < 0$, then $v_x = -1$. Hence Bob can construct a bit-string $\tilde{v}$ given as $\tilde{v}_x = \text{sign}(h(|x\rangle\langle x|)), \forall x = 1, \ldots, d$. Using Equation (S83), we know that at least $d/2$ bits in $\tilde{v}$ will be equal to v.

Because with high probability, $\tilde{v}$ and v has at least $d/2$ bits in common. Fano's inequality tells us that $I(v, \tilde{v}) \geq \Omega(d)$. Because the bit-string $\tilde{v}$ is constructed solely from the training data $\mathcal{T}$. Data processing inequality tells us that $I(v, \tilde{v}) \leq I(v, \mathcal{T})$. Together with Equation (S81), we have

$$N \geq I(v, \mathcal{T}) \geq I(v, \tilde{v}) \geq \Omega(d). \tag{S84}$$

Recall that $d = B/4\epsilon^2$, we have hence obtained the desired result $N \geq \Omega(B/\epsilon^2)$.

9. LIMITATIONS OF QUANTUM KERNEL METHODS

Even though the quantum kernel method saturates the fundamental lower bound $\Omega(\mathrm{Tr}(O^2)/\epsilon^2)$ and can be made formally equivalent to infinite depth quantum neural networks it has a number of limitations that hinder its practical applicability. In this section we construct a simple example where the overhead for using the quantum kernel method is exponential in comparison to trivial classical methods.

Specifically, it has the limitation of closely following this lower bound for any unitary U and observable O. This is not true for other machine learning methods, such as classical neural networks or projected quantum kernel methods. It is possible for classical machine learning methods to learn quantum models with exponentially large $\mathrm{Tr}(O^2)$, which is not learnable by the quantum kernel method. This can already be seen in the numerical experiments given in the main text. In this section, we provide a simple example that allows theoretical analysis to illustrate this limitation.

We consider a simple learning task where the input vector $\mathbf{x} \in \{0, \pi\}^n$. The encoding of the input vector $\mathbf{x}$ to the quantum state space is given as

$$|\mathbf{x}\rangle = \prod_{k=1}^{n} \exp(\mathrm{i} X_k x_k) \left|0^n\right\rangle. \tag{S85}$$

The quantum state $|\mathbf{x}\rangle$ is a computational basis state. We define $\rho(\mathbf{x}) = |\mathbf{x}\rangle\langle\mathbf{x}|$. The quantum model applies a unitary $U = I$, and measures the observable $O = I \otimes \ldots \otimes I \otimes Z$. Hence $f(\mathbf{x}) = \mathrm{Tr}(O\rho(\mathbf{x})) = (2x_n - \pi)$. Notice that for this very simple quantum model, the function $f(\mathbf{x})$ is an extremely simple linear model. Hence a linear regression or a single-layer neural network can learn the function $f(\mathbf{x})$ from training data of size n with high probability.

Despite being a very simple quantum model, the Frobenius norm of the observable $\mathrm{Tr}(O^2)$ is exponentially large, i.e., $\mathrm{Tr}(O^2) = 2^n$. We now show that a quantum kernel method will need a training data of size $N \geq \Omega(2^n)$ to learn this simple function $f(\mathbf{x})$. Suppose we have obtained a training set given as $\{(\mathbf{x}_i, \mathrm{Tr}(O\rho(\mathbf{x}_i))\}_{i=1}^N$ where each $\mathbf{x}_i$ is selected uniformly at random from $\{0, \pi\}^n$. Recall from the analysis in Section 4 A, the function learned by the quantum kernel method will be

$$h^{\mathrm{Q}}(\mathbf{x}) = \min\left(1, \max\left(-1, \sum_{i=1}^{N}\sum_{j=1}^{N} \mathrm{Tr}(\rho(\mathbf{x}_i)\rho(\mathbf{x}))((K^{\mathrm{Q}} + \lambda I)^{-1})_{ij}\, \mathrm{Tr}(O\rho(\mathbf{x}_j))\right)\right), \tag{S86}$$

where $K^Q_{ij} = k^{\mathrm{Q}}(\mathbf{x}_i, \mathbf{x}_j) = \mathrm{Tr}(\rho(\mathbf{x}_i)\rho(\mathbf{x}_j))$. The main problem of the quantum kernel method comes from the precise definition of the kernel function $k(\mathbf{x}_i, \mathbf{x}) = \mathrm{Tr}(\rho(\mathbf{x}_i)\rho(\mathbf{x}))$. For at least $2^n - N$ choices of $\mathbf{x}$, we have $\mathrm{Tr}(\rho(\mathbf{x}_i)\rho(\mathbf{x})) = 0, \forall i = 1, \ldots, N$. This means that for at least $2^n - N$ choices of $\mathbf{x}$, $h^{\mathrm{Q}}(\mathbf{x}) = 0$. However, by construction, $f(\mathbf{x}) \in \{1, -1\}$. Hence the prediction error can be lower bounded by

$$\frac{1}{2^n} \sum_{\mathbf{x} \in \{0,\pi\}^n} |h^{\mathrm{Q}}(\mathbf{x}) - f(\mathbf{x})| \geq 1 - \frac{N}{2^n}. \tag{S87}$$

Therefore, if $N < (1-\epsilon)2^n$, then the prediction error will be greater than ϵ. Hence we need a training set of size $N \geq (1-\epsilon)2^n$ to achieve a prediction error $\leq \epsilon$.

In general, when we place the classical vectors $\mathbf{x}$ into an exponentially large quantum state space, the quantum kernel function $\mathrm{Tr}(\rho(\mathbf{x}_i)\rho(\mathbf{x}_j))$ will be exponentially close to zero for $\mathbf{x}_i \neq \mathbf{x}_j$. In this case K^{Q} will be close to the identity matrix, but $\mathrm{Tr}(\rho(\mathbf{x}_i)\rho(\mathbf{x}))$ will be exponentially small. For a training set of size $N \ll 2^n$, $h^{\mathrm{Q}}(\mathbf{x})$ will be exponentially close to zero similar to the above example. Despite $h^{\mathrm{Q}}(\mathbf{x})$ being exponentially close to zero, if we can distinguish > 0 and < 0, then h^{Q} could still be useful in classification tasks. However, due to the inherent quantum measurement error in evaluating the kernel function $\mathrm{Tr}(\rho(\mathbf{x}_i)\rho(\mathbf{x}_j))$ on a quantum computer, we will need an exponential number of measurements to resolve such an exponentially small difference.

10. PROJECTED QUANTUM KERNEL METHODS

In the main text, we argue that projection back from the quantum space to a classical one in the projected quantum kernel can greatly improve the performance of such methods. There we focused on the simple case of a squared exponential based on reduced 1-particle observables, however this idea is far more general. In this section we explore some of these generalizations including a novel scheme for calculating functions of all powers of RDMs efficiently.

From discussions on the quantum kernel method, we have seen that using the native quantum state space to define the kernel function, e.g., $k(x_i, x_j) = \mathrm{Tr}(\rho(x_i)\rho(x_j))$ can fail to learn even a simple function when the full exponential quantum state space is being used. We have to utilize the entire exponential quantum state space otherwise the quantum machine learning model could be simulated efficiently classically and a large advantage could not be found. In this section, we will detail a set of solutions that project the quantum states back to approximate classical representations and define the kernel function using the classical representation. We refer to these modified quantum kernels as projected quantum kernels. The projected quantum kernels are defined in a classical vector space to circumvent the hardness of learning due to the exponential dimension in quantum Hilbert space. However, projected quantum kernels still use the exponentially large quantum Hilbert space for evaluation and can be hard to simulate classically.

Some simple choices based on reduced density matrices (RDMs) of the quantum state are given below.

1. A linear kernel function using 1-RDMs

$$Q_l^1(x_i, x_j) = \sum_k \mathrm{Tr}\left[\mathrm{Tr}_{m\neq k}[\rho(x_i)]\mathrm{Tr}_{n\neq k}[\rho(x_j)]\right], \tag{S88}$$

   where $\mathrm{Tr}_m \neq k(\rho)$ is the partial trace of the quantum state ρ over all qubits except for the k-th qubit. It could learn any observable that can be written as a sum of one-body terms.

2. A Gaussian kernel function using 1-RDMs

$$Q_g^1(x_i, x_j) = \exp\left(-\gamma \sum_k \left(\mathrm{Tr}_{m\neq k}[\rho(x_i)] - \mathrm{Tr}_{n\neq k}[\rho(x_j)]\right)^2\right), \tag{S89}$$

   where $\gamma > 0$ is a hyper-parameter. It could learn any nonlinear function of the 1-RDMs.

3. A linear kernel using $k-$RDMs

$$Q_l^k(x_i, x_j) = \sum_{K\in S_k(n)} \mathrm{Tr}\left[\mathrm{Tr}_{n\notin K}[\rho(x_i)]\mathrm{Tr}_{m\notin K}[\rho(x_j)]\right] \tag{S90}$$

   where $S_k(n)$ is the set of subsets of k qubits from n, $\mathrm{Tr}_{n\notin K}$ is a partial trace over all qubits not in subset K. It could learn any observable that can be written as a sum of k-body terms.

The above choices have a limited function class that they can learn, e.g., Q_l^1 can only learn observables that are sum of single-qubit observables. It is desirable to define a kernel that can learn any quantum models (e.g., arbitrarily deep quantum neural networks) with sufficient amount of data similar to the original quantum kernel $k^{\mathrm{Q}}(x_i, x_j) = \mathrm{Tr}(\rho(x_i)\rho(x_j))$ as discussed in Supplementary Section 3.

We now define a projected quantum kernel that contains all orders of RDMs. Since all quantum models $f(x) = \mathrm{Tr}(OU\rho(x)U^\dagger)$ are linear functions of the full quantum state, this kernel can learn any quantum models with sufficient data. A k-RDM of a quantum state $\rho(x)$ for qubit indices $(p_1, p_2, \ldots, p_k)$ can be reconstructed by local randomized measurements using the formalism of classical shadows [9]:

$$\rho^{(p_1,p_2,\ldots,p_k)}(x) = \mathbb{E}\left[\otimes_{r=1}^k (3\,|s_{p_r}, b_{p_r}\rangle\langle s_{p_r}, b_{p_r}| - I)\right], \tag{S91}$$

where b_{p_r} is a random Pauli measurement basis X, Y, Z on the p_r-th qubit, and s_{p_r} is the measurement outcome ± 1 on the p_r-th qubit of the quantum state $\rho(x)$ under Pauli basis b_{p_r}. The expectation is taken with respect to the randomized measurement on $\rho(x)$. The inner product of two k-RDMs is equal to

$$\mathrm{Tr}\left[\rho^{(p_1,p_2,\ldots,p_k)}(x_i)\rho^{(p_1,p_2,\ldots,p_k)}(x_j)\right] = \mathbb{E}\left[\Pi_{r=1}^k (9\delta_{s^i_{p_r} s^j_{p_r}}\delta_{b^i_{p_r} b^j_{p_r}} - 4)\right], \tag{S92}$$

where we used the fact that the randomized measurement outcomes for $\rho(x_i)$ and $\rho(x_j)$ are independent. We extend this equation to the case where some indices p_r, p_s coincide. This would only introduce additional features in the feature map $\phi(x)$ that defines the kernel $k(x_i, x_j) = \phi(x_i)^\dagger \phi(x_j)$. The sum of all possible k-RDMs can be written as

$$Q^k(\rho(x_i), \rho(x_j)) = \sum_{p_1=1}^{n} \cdots \sum_{p_k=1}^{n} \mathrm{Tr}\left[\rho^{(p_1,p_2,\ldots,p_k)}(x_i)\rho^{(p_1,p_2,\ldots,p_k)}(x_j)\right] = \mathbb{E}\left[\left(\sum_{p=1}^{n}(9\delta_{s_p^i s_p^j}\delta_{b_p^i b_p^j} - 4)\right)^k\right], \tag{S93}$$

where we used Equation (S92) and linearity of expectation. A kernel function that contains all orders of RDMs can be evaluated as

$$Q_\gamma^\infty(\rho(x_i), \rho(x_j)) = \sum_{k=0}^{\infty} \frac{\gamma^k}{k! n^k} Q^k(\rho(x_i), \rho(x_j)) = \mathbb{E}\exp\left(\frac{\gamma}{n}\sum_{p=1}^{n}(9\delta_{s_p^i s_p^j}\delta_{b_p^i b_p^j} - 4)\right), \tag{S94}$$

where γ is a hyper-parameter. The kernel function $Q_\gamma^\infty(\rho(x_i), \rho(x_j))$ can be computed by performing local randomized measurement on the quantum states $\rho(x_i)$ and $\rho(x_j)$ independently. First, we collect a set of randomized measurement data for $\rho(x_i), \rho(x_j)$ independently:

$$\rho(x_i) \to \{((s_1^{i,r}, b_1^{i,r}), \ldots, (s_n^{i,r}, b_n^{i,r})), \forall r = 1, \ldots, N_s\}, \tag{S95}$$

$$\rho(x_j) \to \{((s_1^{j,r}, b_1^{j,r}), \ldots, (s_n^{j,r}, b_n^{j,r})), \forall r = 1, \ldots, N_s\}, \tag{S96}$$

where N_s is the number of repetition for each quantum state. For each repetition, we will randomly sample a Pauli basis for each qubit and measure that qubit to obtain an outcome ± 1. For the r-th repetition, the Pauli basis in the k-th qubit is given as $b_k^{i,r}$ and the measurement outcome ± 1 is given as $s_k^{i,r}$. Then we compute

$$\frac{1}{N_s(N_s-1)} \sum_{r_1=1}^{N_s} \sum_{\substack{r_2=1 \\ r_2 \neq r_1}}^{N_s} \exp\left(\frac{\gamma}{n}\sum_{p=1}^{n}(9\delta_{s_p^{i,r_1} s_p^{j,r_2}}\delta_{b_p^{i,r_1} b_p^{j,r_2}} - 4)\right) \approx Q_\gamma^\infty(\rho(x_i), \rho(x_j)). \tag{S97}$$

We reuse all pairs of data r_1, r_2 to reduce variance when estimating $Q_\gamma^\infty(\rho(x_i)$, since the resulting estimator would still be equal to the desired quantity in expectation. This technique is known as U-statistics, which is often used to create minimum-variance unbiased estimators. U-statistics is also applied in [9] for estimating Renyi entanglement entropy with high accuracy.

11. SIMPLE AND RIGOROUS QUANTUM ADVANTAGE

In Ref.[10], the authors proposed a machine learning problem based on discrete logarithm which is assumed to be hard for any classical machine learning algorithm, complementing existing work studying learnability in the context of discrete logs [11, 12]. Much of the challenge in their construction [12] was related to technicalities involved in the original quantum kernel approach. Here we present a simple quantum machine learning algorithm using the projected quantum kernel method. The problem is defined as follows, where p is an exponentially large prime number and g is chosen such that computing $\log_g(x)$ in $\mathbb{Z}_p^*$ is classically hard and $\log_g(x)$ is one-to-one.

Definition 1 (Discrete logarithm-based learning problem). *For all input $x \in \mathbb{Z}_p^*$, where $n = \lceil \log_2(p) \rceil$, the output is*

$$y(x) = \begin{cases} +1, & \log_g(x) \in [s, s + \frac{p-3}{2}], \\ -1, & \log_g(x) \notin [s, s + \frac{p-3}{2}], \end{cases} \tag{S98}$$

for some $s \in \mathbb{Z}_p^*$. The goal is to predict $y(x)$ for an input x sampled uniformly from $\mathbb{Z}_p^*$.

Let us consider the most straight-forward feature mapping that maps the classical input x into the quantum state space $|\log_g(x)\rangle$ using Shor's algorithm for computing discrete logarithms [13].

Training the original quantum kernel method using this feature mapping will require training data $\{(x_i, y_i)\}_{i=1}^N$ with N being exponentially large to yield a small prediction error. This is because for a new $x \in \mathbb{Z}_p^*$, such that $\log_g(x) \neq \log_g(x_i), \forall i = 1, \ldots, N$, quantum kernel method will be equivalent to random guessing. Hence the quantum kernel method has to see most of the values in the range of $\log_g(x)$ ($\mathbb{Z}_p^*$) to make accurate predictions. This is the same as the example to demonstrate the limitation of quantum kernel methods in Supplementary Section 9. Since

$\mathbb{Z}_p^*$ is exponentially large, the quantum kernel method has to use an exponentially amount number of data N for this straight-forward feature map. The central problem is that all the inputs x are maximally far apart from one another, and this impedes the ability for quantum kernel methods to generalize.

On the other hand, we can project the quantum feature map $|\log_g(x)\rangle$ back to a classical space, which is now just a number $\log_g(x) \in \mathbb{Z}_p^*$. Recall that $\mathbb{Z}_p^*$ contains all number from $0, \ldots, p-1$, thus we consider mapping x to a real number $z = \log_g(x)/p \in [0, 1)$. Let us define $t = s/p$. In this projected space, we are learning a simple classification problem where $y(z) = +1$ if $z \in [t, t + \frac{p-3}{2p}]$, and $y(z) = -1$ if $z \notin [t, t + \frac{p-3}{2p}]$. We are using a periodic boundary where 0 and 1 are the same point. If $t + \frac{p-3}{2p} < 1$, then there exists some $a, b \in [0, 1)$ and $a < b$, such that $y(z) = +1$, if $a \leq z \leq b$, and $y(z) = -1$, otherwise. In this case we have $y(z) = \mathrm{sign}((b-z)(z-a))$, where $\mathrm{sign}(t) = +1$ if $t \geq 0$, otherwise $\mathrm{sign}(t) = -1$. If $t + \frac{p-3}{2p} \geq 1$, then there exists some $a, b \in [0, 1)$ and $a < b$, such that $y(z) = -1$, if $a \leq z \leq b$, and $y(z) = +1$, otherwise. In this case we have $y(z) = \mathrm{sign}((a-z)(z-b))$. Through this analysis, we can see that we only need to learn a simple quadratic function to perform accurate classification. Hence one could simply define a projected quantum kernel as

$$k^{\mathrm{PQ}}(x_i, x_j) = \left((\log_g(x_i)/p)(\log_g(x_j)/p) + 1\right)^2, \tag{S99}$$

where the division in $(\log_g(x_i)/p)$ is performed as real number in $\mathbb{R}$. This projected quantum kernel can efficiently learn any quadratic function $az^2 + bz + c$ with $z = \log_g(x_i)/p$, hence solving the above learning problem.

Theorem 4 (Corollary 3.19 in [5]). *Let $\mathcal{H}$ be a class of functions taking values in $\{+1, -1\}$ with VC-dimension d. Then with probability $\geq 1 - \delta$ over the sampling of $z_1, \ldots z_N$ from some distribution $\mathcal{D}$, we have*

$$\mathop{\mathbb{E}}_{z\sim\mathcal{D}} I[h(z) \neq y(z)] \leq \frac{1}{N}\sum_{i=1}^{N} I[h(z_i) \neq y(z_i)] + \sqrt{\frac{2d\log(\mathrm{e}N/d)}{N}} + \sqrt{\frac{\log(1/\delta)}{N}}, \tag{S100}$$

for all $h \in \mathcal{H}$, where $I[Statement] = 1$ if Statement is true, otherwise $I[Statement] = 0$.

A simple and rigorous statement could be made by noticing that the VC-dimension [5, 14] for the function class $\{\mathrm{sign}(az^2 + bz + c) | a, b, c \in \mathbb{R}\}$ is 3. Let us apply Theorem 4 with

$$z = \log_g(x)/p \;\text{ and }\; \mathcal{H} = \{\mathrm{sign}(az^2 + bz + c) | a, b, c \in \mathbb{R}\}. \tag{S101}$$

This theorem bounds the prediction error for new inputs z coming from the same distribution as how the training data is sampled. For a given set of training data $(z_i, y(z_i))_{i=1}^N$, we perform a minimization over $a, b, c \in \mathbb{R}$ such that the training error $\frac{1}{N}\sum_{i=1}^N I[h(z_i) \neq y(z_i)]$ is zero. This can be achieved by applying a standard support vector machine algorithm [15] using the above kernel k^{PQ}, because $y(z_i) \in \mathcal{H}$, so one can always fit the training data perfectly. Using Eq. (S100) with $\delta = 0.01$, we can provide a prediction error bound for the trained projected quantum kernel method

$$f_*(x) = h_*(\log_g(x)/p) = h_*(z) = \mathrm{sign}(a_* z^2 + b_* z + c_*). \tag{S102}$$

Because we fit the training data perfectly, we have

$$\frac{1}{N}\sum_{i=1}^{N} I[h_*(z_i) \neq y(z_i)] = 0. \tag{S103}$$

With probability at least 0.99, a projected quantum kernel method $f_*(x) = h_*(\log_g(x)/p)$ that perfectly fit a data set of size $N = \mathcal{O}(\log(1/\epsilon)/\epsilon^2)$ has a prediction error

$$\mathop{\mathbb{P}}_{x\sim\mathbb{Z}_p^*}[f(x) \neq y(x)] \leq \epsilon. \tag{S104}$$

This concludes the proof showing that the discrete logarithm-based learning problem can be solved with a projected quantum kernel method using a sample complexity independent of the input size n.

Despite the limitations of the quantum kernel method, the authors in [10] have shown that a clever choice of feature mapping $x \to \rho(x)$ would also allow quantum kernels $\mathrm{Tr}(\rho(x_i)\rho(x_j))$ to predict well in this learning problem.

12. DETAILS OF NUMERICAL STUDIES

Here we give the complete details for the numerical studies presented in the main text. For the input distribution, we focused on the fashion MNIST dataset [16]. We use principal component analysis (PCA) provided by scikit-learn [17] to map each image (28×28 grayscale) into classical vectors $\mathbf{x}_i \in \mathbb{R}^n$, where n is the number of principal components. After PCA, we normalize the vectors $\mathbf{x}_i$ such that each dimension is centered at zero and the standard deviation is one. Finally, we sub-sample 800 data points from the dataset without replacement.

A. Embedding classical data into quantum states

The three approaches for embedding classical vectors $\mathbf{x}_i \in \mathbb{R}^n$ into quantum states $|\mathbf{x}_i\rangle$ are given below.

- **E1**: Separable encoding or qubit rotation circuit. This is a common choice in literature, e.g., see [18, 19].

$$|\mathbf{x}_i\rangle = \bigotimes_{j=1}^{n} \mathrm{e}^{-\mathrm{i} X_j x_{ij}} |0^n\rangle , \tag{S105}$$

where x_{ij} is the j-th entry of the n-dim. vector $\mathbf{x}_i$, X_j is the Pauli-X operator acting on the j-th qubit.

- **E2**: IQP-style encoding circuit. This is an embedding proposed in [20] that suggests a quantum advantage.

$$|\mathbf{x}_i\rangle = U_Z(\mathbf{x}_i) H^{\otimes n} U_Z(\mathbf{x}_i) H^{\otimes n} |0^n\rangle , \tag{S106}$$

where $H^{\otimes n}$ is the unitary that applies Hadamard gates on all qubits in parallel, and

$$U_Z(\mathbf{x}_i) = \exp\left(\sum_{j=1}^{n} x_{ij} Z_j + \sum_{j=1}^{n} \sum_{j'=1}^{n} x_{ij} x_{ij'} Z_j Z_{j'} \right) , \tag{S107}$$

with Z_j defined as the Pauli-Z operator acting on the j-th qubit. In the original proposal [20], $x \in [0, 2\pi]^n$, and they used $U_Z(\mathbf{x}_i) = \exp\left(\sum_{j=1}^{n} x_{ij} Z_j + \sum_{j=1}^{n} \sum_{j'=1}^{n} (\pi - x_{ij})(\pi - x_{ij'}) Z_j Z_{j'} \right)$ instead. Here, due to the data pre-processing steps, $\mathbf{x}$ will be centered around 0 with a standard deviation of 1, hence we made the equivalent changes to the definition of $U_Z(\mathbf{x}_i)$.

- **E3**: A Hamiltonian evolution ansatz. This ansatz has been explored in the literature [21–23] for quantum many-body problems. We consider a Trotter formula with T Trotter steps (we choose $T = 20$) for evolving an 1D-Heisenberg model with interactions given by the classical vector $\mathbf{x}_i$ for a time t proportional to the system size (we choose $t = n/3$).

$$|\mathbf{x}_i\rangle = \left(\prod_{j=1}^{n} \exp\left(-\mathrm{i} \frac{t}{T} x_{ij} \left(X_j X_{j+1} + Y_j Y_{j+1} + Z_j Z_{j+1} \right) \right) \right)^T \bigotimes_{j=1}^{n+1} |\psi_j\rangle , \tag{S108}$$

where X_j, Y_j, Z_j are the Pauli operators for the j-th qubit and $|\psi_j\rangle$ is a Haar-random single-qubit quantum state. We sample and fix the Haar-random quantum states $|\psi_j\rangle$ for every qubit.

B. Definition of original and projected quantum kernels

We use Tensorflow-Quantum [24] for implementing the original/projected quantum kernel methods. This is done by performing quantum circuit simulation for the above embeddings and computing the kernel function $k(\mathbf{x}_i, \mathbf{x}_j)$. For quantum kernel, we store the quantum states $|\mathbf{x}_i\rangle$ as explicit amplitude vectors and compute the squared inner product

$$k^{\mathrm{Q}}(\mathbf{x}_i, \mathbf{x}_j) = |\langle \mathbf{x}_i | \mathbf{x}_j \rangle|^2. \tag{S109}$$

On actual quantum computers, we obtain the quantum kernel by measuring the expectation of the observable $|0^n\rangle\langle 0^n|$ on the quantum state $U_{\mathrm{emb}}(\mathbf{x}_j)^\dagger U_{\mathrm{emb}}(\mathbf{x}_i) |0^n\rangle$. For projected quantum kernel, we use the kernel function

$$k^{\mathrm{PQ}}(\mathbf{x}_i, \mathbf{x}_j) = \exp\left(-\gamma \sum_k \sum_{P \in \{X,Y,Z\}} \left(\mathrm{Tr}(P \rho(\mathbf{x}_i)_k) - \mathrm{Tr}(P \rho(\mathbf{x}_j)_k) \right)^2 \right) , \tag{S110}$$

where P is a Pauli matrix and $\gamma > 0$ is a hyper-parameter chosen to maximize prediction accuracy. We compute the kernel matrix $K \in \mathbb{R}^{N \times N}$ with $K_{ij} = k(\mathbf{x}_i, \mathbf{x}_j)$ using the sub-sampled dataset with $N = 800$ for both the original/projected quantum kernel.

C. Dimension and geometric difference

Following the discussion in Supplementary Section 6 B, the approximate dimension of the original/projected quantum space is computed by

$$\sum_{k=1}^{N} \left(\frac{1}{N-k} \sum_{l=k}^{N} t_l \right), \tag{S111}$$

where $N = 800$ and $t_1 \geq t_2 \geq \ldots \geq t_N$ are the singular values of the kernel matrix $K \in \mathbb{R}^{N \times N}$. Based on the discussion in Supplementary Section 6 C, we report the minimum geometric difference g of the original/projected quantum space (we refer to both the original/projected quantum kernel matrix as $K^{\mathrm{P/Q}}$)

$$g_{\text{gen}} = \sqrt{\left\| \sqrt{K^{\mathrm{P/Q}}} \sqrt{K^{\mathrm{C}}} \left(K^{\mathrm{C}} + \lambda I \right)^{-2} \sqrt{K^{\mathrm{C}}} \sqrt{K^{\mathrm{P/Q}}} \right\|_{\infty}}, \tag{S112}$$

under a condition for having a small training error

$$g_{\text{tra}} = \lambda \sqrt{\left\| \sqrt{K^{\mathrm{P/Q}}} (K^{\mathrm{C}} + \lambda I)^{-2} \sqrt{K^{\mathrm{P/Q}}} \right\|_{\infty}} < 0.045. \tag{S113}$$

The actual value of g will depend on the list of choices for λ and classical kernels K^{C}. We consider the following list of λ

$$\lambda \in \{0.00001, 0.0001, 0.001, 0.01, 0.025, 0.05, 0.1\}, \tag{S114}$$

and classical kernel matrix K^{C} being the linear kernel $k^{\ell}(\mathbf{x}_i, \mathbf{x}_j) = \mathbf{x}_i^{\dagger} \mathbf{x}_j$ or the Gaussian kernel $k^{\gamma}(\mathbf{x}_i, \mathbf{x}_j) = \exp(-\gamma \left\| \mathbf{x}_i - \mathbf{x}_j \right\|^2)$ with hyper-parameter γ from the list

$$\gamma \in \{0.25, 0.5, 1.0, 2.0, 4.0, 8.0, 16.0, 32.0, 64.0\} / (n \, \mathrm{Var}[x_{ik}]) \tag{S115}$$

for estimating the minimum geometric difference. $\mathrm{Var}[x_{ik}]$ is the variance of all the coordinates $k = 1, \ldots, n$ from all the data points $x_1, \ldots, x_N$. One could add more choices of regularization parameters λ or classical kernel functions, such as using polynomial kernels or neural tangent kernels, which are equivalent to training neural networks with large hidden layers (a package, called Neural Tangents [25], is available for use). This will provide a smaller geometric difference with the quantum state space, but all theoretical predictions remain unchanged.

D. Datasets

We include a variety of classical and quantum data sets.

1. **Dataset (C)**: For the original classical image recognition data set, i.e., Dataset (C) in Figure 3(b), we choose two classes, dresses (class 3) and shirts (class 6), to form a binary classification task. The prediction error (between 0.0 and 1.0) is equal to the portion of data that are incorrectly labeled.

2. **Dataset (Q, E1/E2/E3)**: For the quantum data sets in Figure 3(b), we consider the following quantum neural network

$$U_{\text{QNN}} = \left(\prod_{j=1}^{n} \exp\left(-\mathrm{i} \frac{t}{T} J_j \left(X_j X_{j+1} + Y_j Y_{j+1} + Z_j Z_{j+1} \right) \right) \right)^T, \tag{S116}$$

where we choose $T = t = 10$ and $J_j \in \mathbb{R}$ are randomly sampled from the Gaussian distribution with mean 0 and standard deviation 1. We measure Z_1 after the quantum neural network, hence the resulting function is

$$f(\mathbf{x}) = \mathrm{Tr}(Z_1 U_{\text{QNN}} \left|\mathbf{x}\right\rangle\!\left\langle\mathbf{x}\right| U_{\text{QNN}}^{\dagger}). \tag{S117}$$

The mapping from $\mathbf{x}$ to $|\mathbf{x}\rangle$ depends on the feature embedding (E1, E2, or E3) discussed in Section 12 A. A different embedding $|\mathbf{x}\rangle$ corresponds to a different funtion $f(\mathbf{x})$, and hence would result in a different dataset. The prediction error for these datasets are the average absolute error with $f(\mathbf{x})$.

3. **Engineered datasets**: In Figure 4, we consider datasets that are engineered to saturate the potential of a quantum ML model. Given the choice of classical kernel K^{C} that has the smallest geometric difference g with a quantum ML model K^{Q}, we can create a data set that saturates $s_{\mathrm{C}} = g^2 s_{\mathrm{Q}}$ following the procedure in Supplementary Section 7. In particular, we construct the dataset such that $s_{\mathrm{Q}} = 1$ and $s_{\mathrm{C}} = g^2$. We compute the eigenvector $\mathbf{v}$ corresponding to the maximum eigenvalue of

$$\sqrt{K^{\mathrm{Q}}}\sqrt{K^{\mathrm{C}}}\left(K^{\mathrm{C}} + \lambda I\right)^{-2}\sqrt{K^{\mathrm{C}}}\sqrt{K^{\mathrm{Q}}} \tag{S118}$$

and construct $\mathbf{y}' = \sqrt{K^{\mathrm{Q}}}\mathbf{v} \in \mathbb{R}^N$. y_i' corresponds to a real number for data point $\mathbf{x}_i$. Finally we define the label of input data point $\mathbf{x}_i$ as

$$y_i = \begin{cases} \mathrm{sign}(y_i'), & \text{with prob. } 0.9, \\ \text{random} \pm 1, & \text{with prob. } 0.1. \end{cases} \tag{S119}$$

This data set will show the maximal separation between quantum and classical ML model. The plots in Figure 4 uses engineered datasets generated by saturating the geometric difference of classical ML models and quantum ML models based on projected quantum kernels in Equation (S110) under different embeddings (E1, E2, and E3). In Supplementary Figure 2, we show the results for quantum ML models based on the original quantum kernels.

E. Classical machine learning models

We present the list of classical machine learning models that we compared with. We used scikit-learn [17] for training the classical ML models.

- Neural network: We perform a grid search over two-layer feedforward neural networks with hidden layer size

$$h \in \{10, 25, 50, 75, 100, 125, 150, 200\}. \tag{S120}$$

For classification, we use MLPClassifier. For regression, we use MLPRegressor.

- Linear kernel method: We perform a grid search over the regularization parameter

$$C \in \{0.006, 0.015, 0.03, 0.0625, 0.125, 0.25, 0.5, 1.0, 2.0, 4.0, 8.0, 16.0, 32.0, 64.0, 128.0, 256, 512, 1024\}. \tag{S121}$$

For classification, we use SVC with linear kernel. For regression, we choose the best between SVR and KernelRidge (both using linear kernel).

- Gaussian kernel method: We perform a grid search over the regularization parameter

$$C \in \{0.006, 0.015, 0.03, 0.0625, 0.125, 0.25, 0.5, 1.0, 2.0, 4.0, 8.0, 16.0, 32.0, 64.0, 128.0, 256, 512, 1024\}. \tag{S122}$$

and kernel hyper-parameter

$$\gamma \in \{0.25, 0.5, 1.0, 2.0, 3.0, 4.0, 5.0, 20.0\}/(n\,\mathrm{Var}[x_{ik}]). \tag{S123}$$

$\mathrm{Var}[x_{ik}]$ is the variance of all the coordinates $k = 1, \ldots, n$ from all the data points $\mathbf{x}_1, \ldots, \mathbf{x}_N$. For classification, we use SVC with RBF kernel (equivalent to Gaussian kernel). For regression, we choose the best between SVR and KernelRidge (both using RBF kernel).

- Random forest: We perform a grid search over the individual tree depth

$$\text{max\_depth} \in \{2, 3, 4, 5\}, \tag{S124}$$

and number of trees

$$\text{n\_estimators} \in \{25, 50, 100, 200, 500\}. \tag{S125}$$

For classification, we use RandomForestClassifier. For regression, we use RandomForestRegressor.

- Gradient boosting: We perform a grid search over the individual tree depth

$$\text{max\_depth} \in \{2, 3, 4, 5\}, \tag{S126}$$

and number of trees

$$\text{n\_estimators} \in \{25, 50, 100, 200, 500\}. \tag{S127}$$

For classification, we use GradientBoostingClassifier. For regression, we use GradientBoostingRegressor.

- Adaboost: We perform a grid search over the number of estimators

$$\text{n\_estimators} \in \{25, 50, 100, 200, 500\}. \tag{S128}$$

For classification, we use AdaBoostClassifier. For regression, we use AdaBoostRegressor.

F. Quantum machine learning models

For training quantum kernel methods, we use the kernel function $k^{\mathrm{Q}}(\mathbf{x}_i, \mathbf{x}_j) = \mathrm{Tr}(\rho(\mathbf{x}_i)\rho(\mathbf{x}_j))$. For classification, we use SVC with the quantum kernel. For regression, we choose the best between SVR and KernelRidge (both using the quantum kernel). We perform a grid search over

$$C \in \{0.006, 0.015, 0.03, 0.0625, 0.125, 0.25, 0.5, 1.0, 2.0, 4.0, 8.0, 16.0, 32.0, 64.0, 128.0, 256, 512, 1024\}. \tag{S129}$$

For training projected quantum kernel methods, we use the kernel function

$$k^{\mathrm{PQ}}(\mathbf{x}_i, \mathbf{x}_j) = \exp\left(-\gamma \sum_k \sum_{P \in \{X,Y,Z\}} \left(\mathrm{Tr}(P\rho(\mathbf{x}_i)_k) - \mathrm{Tr}(P\rho(\mathbf{x}_j)_k)\right)^2\right), \tag{S130}$$

where P is a Pauli matrix. For classification, we use SVC with the projected quantum kernel $k^{\mathrm{PQ}}(\mathbf{x}_i, \mathbf{x}_j)$. For regression, we choose the best between SVR and KernelRidge (both using the projected quantum kernel $k^{\mathrm{PQ}}(\mathbf{x}_i, \mathbf{x}_j)$). We perform a grid search over

$$C \in \{0.006, 0.015, 0.03, 0.0625, 0.125, 0.25, 0.5, 1.0, 2.0, 4.0, 8.0, 16.0, 32.0, 64.0, 128.0, 256, 512, 1024\}. \tag{S131}$$

and kernel hyper-parameter

$$\gamma \in \{0.25, 0.5, 1.0, 2.0, 3.0, 4.0, 5.0, 20.0\}/(n\,\mathrm{Var}[\mathrm{Tr}(P\rho(\mathbf{x}_i)_k)]). \tag{S132}$$

$\mathrm{Var}[\mathrm{Tr}(P\rho(\mathbf{x}_i)_k)]$ is the variance of $\mathrm{Tr}(P\rho(\mathbf{x}_i)_k)$ for all $P \in \{X, Y, Z\}$, all coordinates $k = 1, \ldots, n$, and all data points $x_1, \ldots, x_N$. We report the prediction performance under the best hyper-parameter for all classical and quantum machine learning models.

13. ADDITIONAL NUMERICAL EXPERIMENTS

In the main text, we have presented engineered data sets to saturate the geometric inequality $s_{\mathrm{C}} \leq g(\mathrm{C}||\mathrm{PQ})^2 s_{\mathrm{PQ}}$ between classical ML and projected quantum kernel. As an additional experiment to see if the same approach can work with the original quantum kernel method, we can create similar engineered data sets that saturate the geometric inequality between classical ML and quantum kernel The result is given in Supplementary Figure 2. We can see that due to the large dimension d and small geometric difference $g(\mathrm{C}||\mathrm{Q})$ between classical ML and quantum kernel at large system size, there are no obvious advantage even for this best-case scenario. Interestingly, we see some advantage of projected quantum kernel over classical ML even when this data set is not constructed for projected quantum kernel.

In Supplementary Figure 3, we show the prediction performance for learning a quantum neural network under a wide range for the number of training data N. We can see that there is a non-trivial advantage for small training size $N = 100$ when comparing projected quantum kernel and the best classical ML model. However, as training size N increases, every model will improve and the prediction advantage will shrink.

In Supplementary Figure 4, we compare the prediction error bound $s_K(N)$ for classical kernel methods and the prediction performance of the best classical ML model (including a variety of classical ML models in Section 12 E).

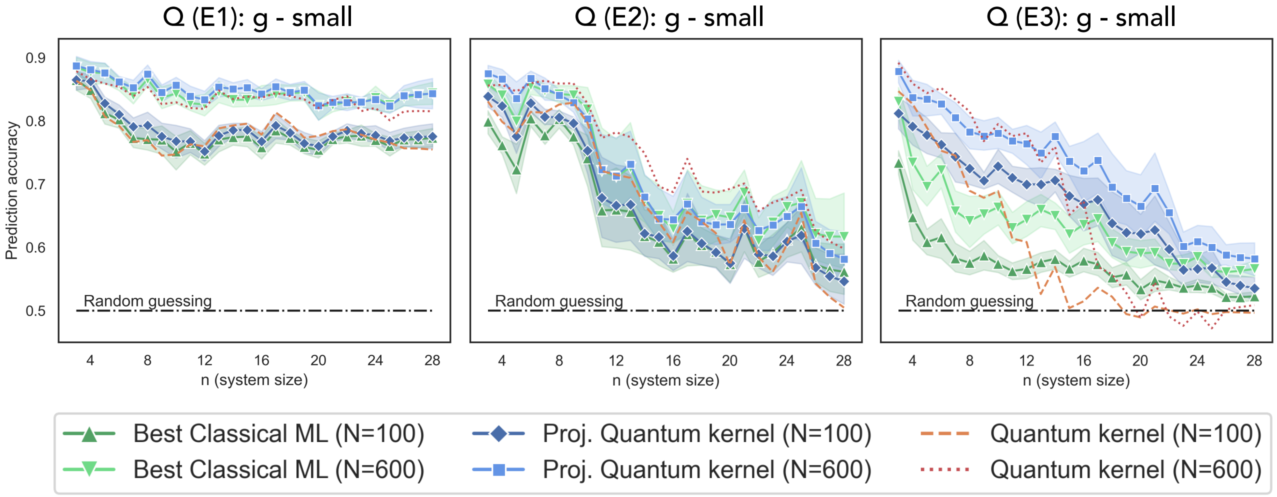

Supplementary Figure 2. Prediction accuracy (higher the better) on engineered data sets. A label function is engineered to match the geometric difference $g(\mathrm{C}||\mathrm{QK})$ between the original quantum kernel and classical approaches. No substantial advantage is found using quantum kernel methods at large system size due to the small geometric difference $g(\mathrm{C}||\mathrm{QK})$. We consider the best performing classical ML models among Gaussian SVM, linear SVM, Adaboost, random forest, neural networks, and gradient boosting.

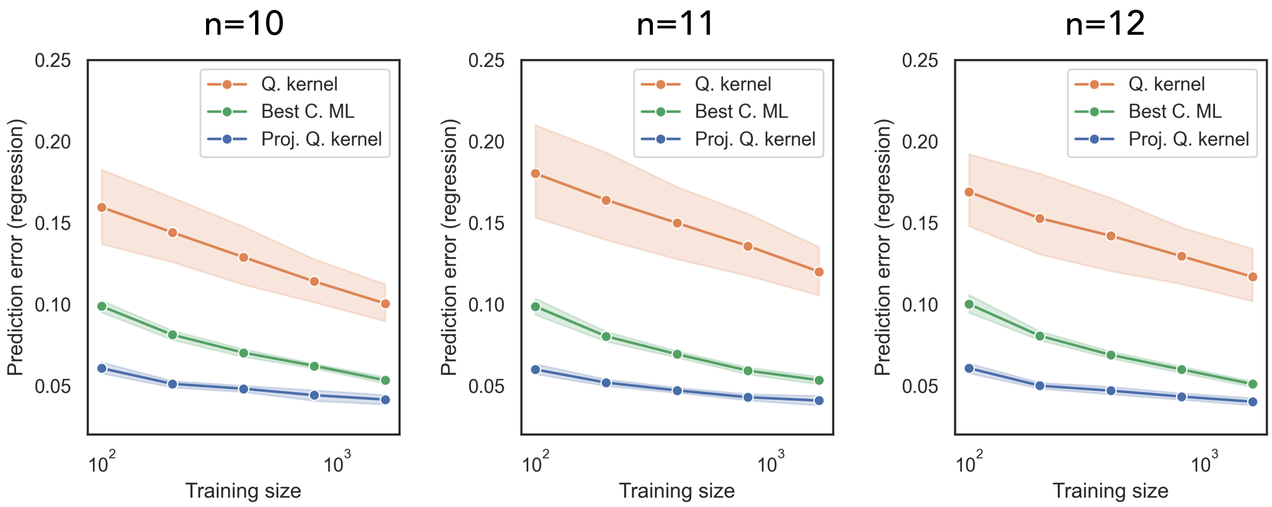

Supplementary Figure 3. Prediction error (lower the better) on quantum data set (E2) over different training set size N. We can see that as the number of data increases, every model improves and the separation between them decreases.

To be more precise, we consider different classical kernel functions and different regularization parameter λ. Then we compute

$$s_{K,\lambda}(N) = \sqrt{\frac{\lambda^2 \sum_{i=1}^N \sum_{j=1}^N ((K+\lambda I)^{-2})_{ij} y_i y_j}{N}} + \sqrt{\frac{\sum_{i=1}^N \sum_{j=1}^N ((K+\lambda I)^{-1} K (K+\lambda I)^{-1})_{ij} y_i y_j}{N}}. \quad \text{(S133)}$$

This is a generalization of $s_K(N)$ described in the main text, where we consider regularized classical kernel methods with a regularization parameter λ to improve generalization performance (setting $\lambda = 0$ reduces to $s_K(N)$ given in the main text). See Section 4 for a detailed proof of an upper bound to the prediction error (note that the output label $y_i = \mathrm{Tr}(O^U \rho(\mathbf{x}_i))$). We can see that while the prediction error bound and the actual prediction error has a non-negligible gap, the two figures follow a similar trend. When the prediction error bound is small, the prediction error of the best classical ML is also fairly small (and vice versa). It shows that $s_{K,\lambda}(N)$ is a good predictive metric

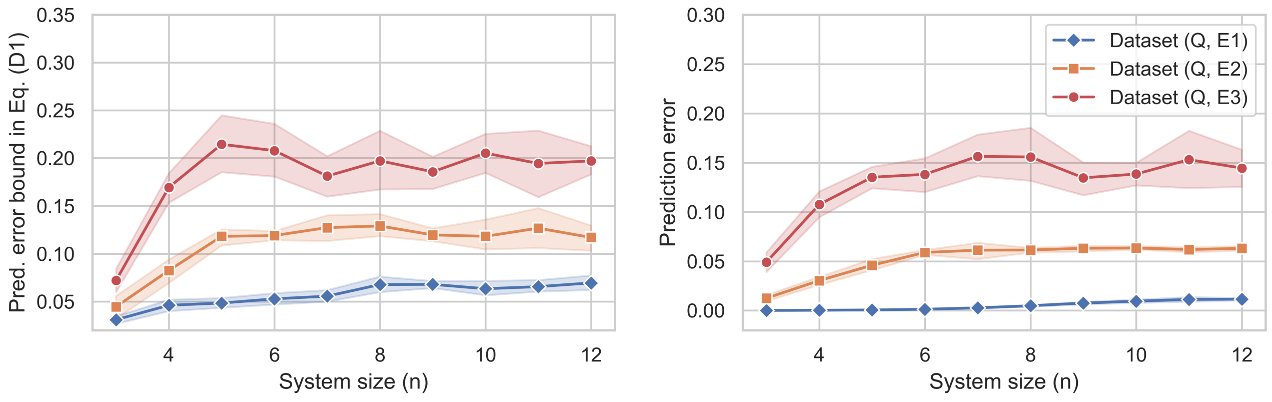

Supplementary Figure 4. A comparison between the prediction error bound based on classical kernel methods (see Eq. (S10)) and the prediction performance of the best classical ML model on the three quantum datasets. We consider the best performing classical ML models among Gaussian SVM, linear SVM, Adaboost, random forest, neural networks, and gradient boosting. While the prediction error bound is an upper bound to the actual prediction error, the trends are very similar (a large prediction error bound gives a large prediction error).

for whether a classical ML model can learn to predict outputs from a quantum computation model.

[1] J. A. Suykens and J. Vandewalle, "Least squares support vector machine classifiers," Neural processing letters **9**, 293 (1999).
[2] E. A. Nadaraya, "On estimating regression," Theory of Probability & Its Applications **9**, 141 (1964).
[3] N. S. Altman, "An introduction to kernel and nearest-neighbor nonparametric regression," The American Statistician **46**, 175 (1992).
[4] A. Jacot, F. Gabriel, and C. Hongler, "Neural tangent kernel: Convergence and generalization in neural networks," arXiv preprint arXiv:1806.07572 (2018).
[5] M. Mohri, A. Rostamizadeh, and A. Talwalkar, *Foundations of machine learning* (2018).
[6] A. Krogh and J. A. Hertz, "A simple weight decay can improve generalization," in *Advances in neural information processing systems* (1992) pp. 950–957.
[7] C. Cortes and V. Vapnik, "Support-vector networks," Machine learning **20**, 273 (1995).
[8] J. Haah, A. W. Harrow, Z. Ji, X. Wu, and N. Yu, "Sample-optimal tomography of quantum states," IEEE Transactions on Information Theory **63**, 5628 (2017).
[9] H.-Y. Huang, R. Kueng, and J. Preskill, "Predicting many properties of a quantum system from very few measurements," Nat. Phys. (2020).
[10] Y. Liu, S. Arunachalam, and K. Temme, "A rigorous and robust quantum speed-up in supervised machine learning," arXiv preprint arXiv:2010.02174 (2020).
[11] R. A. Servedio and S. J. Gortler, "Equivalences and separations between quantum and classical learnability," SIAM Journal on Computing **33**, 1067 (2004).
[12] R. Sweke, J.-P. Seifert, D. Hangleiter, and J. Eisert, "On the quantum versus classical learnability of discrete distributions," arXiv preprint arXiv:2007.14451 (2020).
[13] M. A. Nielsen and I. L. Chuang, "Quantum computation and quantum information," .
[14] A. Blumer, A. Ehrenfeucht, D. Haussler, and M. K. Warmuth, "Learnability and the vapnik-chervonenkis dimension," Journal of the ACM (JACM) **36**, 929 (1989).
[15] C.-C. Chang and C.-J. Lin, "Libsvm: A library for support vector machines," ACM transactions on intelligent systems and technology (TIST) **2**, 1 (2011).
[16] H. Xiao, K. Rasul, and R. Vollgraf, "Fashion-mnist: a novel image dataset for benchmarking machine learning algorithms," arXiv preprint arXiv:1708.07747 (2017).
[17] L. Buitinck, G. Louppe, M. Blondel, F. Pedregosa, A. Mueller, O. Grisel, V. Niculae, P. Prettenhofer, A. Gramfort, J. Grobler, R. Layton, J. VanderPlas, A. Joly, B. Holt, and G. Varoquaux, "API design for machine learning software: experiences from the scikit-learn project," in *ECML PKDD Workshop: Languages for Data Mining and Machine Learning* (2013) pp. 108–122.
[18] M. Schuld and N. Killoran, "Quantum machine learning in feature hilbert spaces," Physical review letters **122**, 040504 (2019).
[19] A. Skolik, J. R. McClean, M. Mohseni, P. van der Smagt, and M. Leib, "Layerwise learning for quantum neural networks,"

arXiv preprint arXiv:2006.14904 (2020).

[20] V. Havlíček, A. D. Córcoles, K. Temme, A. W. Harrow, A. Kandala, J. M. Chow, and J. M. Gambetta, "Supervised learning with quantum-enhanced feature spaces," Nature **567**, 209 (2019).

[21] D. Wecker, M. B. Hastings, and M. Troyer, "Progress towards practical quantum variational algorithms," Physical Review A **92**, 042303 (2015).

[22] C. Cade, L. Mineh, A. Montanaro, and S. Stanisic, "Strategies for solving the fermi-hubbard model on near-term quantum computers," arXiv preprint arXiv:1912.06007 (2019).

[23] R. Wiersema, C. Zhou, Y. de Sereville, J. F. Carrasquilla, Y. B. Kim, and H. Yuen, "Exploring entanglement and optimization within the hamiltonian variational ansatz," arXiv preprint arXiv:2008.02941 (2020).

[24] M. Broughton, G. Verdon, T. McCourt, A. J. Martinez, J. H. Yoo, S. V. Isakov, P. Massey, M. Y. Niu, R. Halavati, E. Peters, *et al.*, "Tensorflow quantum: A software framework for quantum machine learning," arXiv preprint arXiv:2003.02989 (2020).

[25] R. Novak, L. Xiao, J. Hron, J. Lee, A. A. Alemi, J. Sohl-Dickstein, and S. S. Schoenholz, "Neural tangents: Fast and easy infinite neural networks in python," in *International Conference on Learning Representations* (2020).
